# Marine Natural Compound (Neviotin A) Displays Anticancer Efficacy by Triggering Transcriptomic Alterations and Cell Death in MCF-7 Cells

**DOI:** 10.3390/molecules28176289

**Published:** 2023-08-28

**Authors:** Quaiser Saquib, Stefan Schwaiger, Mostafa Alilou, Sarfaraz Ahmed, Maqsood A. Siddiqui, Javed Ahmad, Mohammad Faisal, Eslam M. Abdel-Salam, Rizwan Wahab, Adnan J. Al-Rehaily, Hermann Stuppner, Abdulaziz A. Al-Khedhairy

**Affiliations:** 1 Chair for DNA Research, Zoology Department, College of Sciences, King Saud University, P.O. Box 2455, Riyadh 11451, Saudi Arabia; maqsoodahmads@gmail.com (M.A.S.); javedbiochem@gmail.com (J.A.); rizwannano@gmail.com (R.W.); kedhairy@ksu.edu.sa (A.A.A.-K.); 2 Institute of Pharmacy/Pharmacognosy, Center for Molecular Biosciences Innsbruck, University of Innsbruck, Innrain 80/82, 6020 Innsbruck, Austria; stefan.schwaiger@uibk.ac.at (S.S.); mostafa.alilou@uibk.ac.at (M.A.); hermann.stuppner@uibk.ac.at (H.S.); 3 Department of Pharmacognosy, College of Pharmacy, King Saud University, P.O. Box 2457, Riyadh 11451, Saudi Arabia; ahmsarfaraz@gmail.com (S.A.); ajalreha@ksu.edu.sa (A.J.A.-R.); 4 Department of Botany & Microbiology, College of Sciences, King Saud University, P.O. Box 2455, Riyadh 11451, Saudi Arabia; faisalm15@yahoo.com (M.F.); eabdelsalam@hotmail.com (E.M.A.-S.)

**Keywords:** marine natural product, sponge, apoptosis, anticancer drug, Neviotin A, cell death, ROS, breast cancer

## Abstract

We investigated the anticancer mechanism of a chloroform extract of marine sponge (*Haliclona fascigera*) (sample C) in human breast adenocarcinoma (MCF-7) cells. Viability analysis using MTT and neutral red uptake (NRU) assays showed that sample C exposure decreased the proliferation of cells. Flow cytometric data exhibited reactive oxygen species (ROS), nitric oxide (NO), dysfunction of mitochondrial potential, and apoptosis in sample C-treated MCF-7 cells. A qPCR array of sample C-treated MCF-7 cells showed crosstalk between different pathways of apoptosis, especially *BIRC5*, *BCL2L2*, and *TNFRSF1A* genes. Immunofluorescence analysis affirmed the localization of p53, bax, bcl2, MAPKPK2, PARP-1, and caspase-3 proteins in exposed cells. Bioassay-guided fractionation of sample C revealed Neviotin A as the most active compound triggering maximum cell death in MCF-7, indicating its pharmacological potency for the development of a drug for the treatment of human breast cancer.

## 1. Introduction

Of the Earth’s surface, 70% is covered by the world’s oceans, and 95% of the oceans’ tropical biosphere contains extraordinary diversity in both macro and microscopic organisms [1,2]. It has been estimated that nearly 1 × 10^9^ unicellular and 1 × 10^6^ multicellular organisms, including plants and animals, are accommodated in the oceans [3]. In particular, marine reefs represent a densely crowded ecosystem, providing shelter to >1000 species/m^2^; consequently, fierce competition for food and space exists among its inhabitants [2]. Such conditions have compelled organisms to evolve biochemical and physiological mechanisms for producing secondary metabolites for protection, reproduction, communication, and infection [4].

The adapted organisms generally produce alkaloids, polypeptides, terpenoids, sugars, shikimic acid derivatives, and a range of further metabolites, commonly subsumed as marine natural products (MNPs) [5]. In the quest for safe and effective pharmaceutical drugs, people rely on natural resources; nearly 50% of drugs are either extracted from natural resources or synthesized using natural products as a starting framework. During the span of a thousand years, these natural products possessed unique chemical properties that proceeded to gain drug-like properties [6]. More than 30,000 natural products from marine resources have been isolated so far, exemplifying the importance of the marine biosphere as a reservoir of bioactive compounds that offer varying types of active drugs [7]. In view of the growing demand for mining and data sharing of MNPs, a comprehensive database has recently been developed that contains information about over 30,000 MNPs [7]. Exploration of the marine world has led to the discovery of several approved anticancer drugs, including cytarabine, ecteinascidin 743, brentuximab vedotin, dolastatin 10, and didemnin B [6,8]. Recent approaches have unraveled the efficacy of marine peptides or small peptides as novel therapeutic candidates for antimicrobial and cancer therapy [8,9,10,11]. Nanoparticles developed through green approaches by using bioactive marine compounds have opened a new therapeutic avenue for the delivery of cancer drugs and their antitumor properties [12,13,14]. In 2023, the American Cancer Society expected 1,958,310 new cases of cancer that could cause 609,820 deaths (male and female); within this estimation, 300,590 new cases of breast cancer could account for 43,700 deaths [15]. In this line, marine peptides (hemiasterlin and eribulin mesylate) are approved by the US FDA for the treatment of breast cancer [8,16,17].

During the last 50 years, marine sponges have been regarded as goldmines owing to their diverse range of secondary metabolites. More than 5300 MNPs have been discovered in sponges, and many more are added every year [18]. In 2016, 224 new marine compounds were reported from sponges compared to 291 in 2015 [19]. Consequently, sponges have great potential to provide therapeutic remedies against varying diseases [20]. Plakdiepoxide isolated from the Chinese sponge *Plakortis simplex* was found to activate PPAR-*α* and PPAR-*γ* selectively and may be considered the lead for the treatment of type II diabetes [21]. The two novel scalarane sesterterpenoids isolated from sponge (*Carteriospongia* sp.) induced apoptotic cell death by inhibiting topoisomerase IIa expression, mitochondrial membrane potential, heat shock protein 90, and ROS formation in leukemia (Molt 4) cells [22]. A crude extract of sponge (*Phorbas* sp.) exhibited the presence of eight new sesterterpenoids found to activate HIV proviral gene expression via induction of protein kinase C signaling [23]. The natural isolates of callipeltins C and H from sponge (*Latrunculia* sp.) exhibited their anticancer activity by inhibiting the growth of HeLa cells, indicating their low dose range (15%) bioactivity was due to low levels (~15%) of callipeltins [24]. Pyrroloiminoquinone metabolites from *Latrunculia* sp. inhibit angiogenesis in HCT116 colon cancer cells via induction of hypoxia-inducible factor-1a (*HIF-1a*) [25]. Nrf2-mediated antioxidant improvement in neuronal cells (SHSY5Y) has been found in the extracts of marine sponge *Zyzzya* sp. [26]. DNA methyltransferase 1-targeted apoptosis has been reported for isostularin-3 isolated from sponge (*Aplysina aerophoba*), rendering it a new anticancer drug [27]. A549 lung cells exposed to extracts of *Haliclona* sp. exhibited apoptosis induction via the JNKp53 pathway and caspase-8 activation [28]. In the same line, brominated acetylenic hydrocarbon isolated from *Haliclona* sp. exhibited anticancer effects against MCF-7 cells [29]. Wild-type p53 and p53 knockout HCT116 cells, when exposed to extracts of sponge (*Lipastrotethya* sp.), exhibited apoptotic cell death in the wild type, while the knockout cells demonstrated autophagy induction, showing reductions in mTOR, Bcl-2 and increased levels of Beclin-1 and LC3-II protein [30]. Despite the diversity of bioactive compounds in marine sponges, especially the marine sponges from the Red Sea, they are rarely explored for retrieving anticancer compounds that can kill breast cancer cells (MCF-7). In this line, we found a single report in which three acetylenic brominated derivatives isolated from *Haliclona* sp. of the Red Sea, Jeddah, Saudi Arabia, showed cytotoxic effects in MCF-7 cells [29]. However, solely based on the cytotoxicity screening tests, the isolated compounds were attributed as anticancer entities, while no evidence of the mechanism of anticancer effects has been specified. To plug the data gap, our novel study provides a mechanism-based anticancer investigation in breast cancer cells (MCF-7) using Neviotin A isolated from the chloroform extract (sample C) of *Haliclona fascigera* from the Red Sea, Saudi Arabia. We used a transcriptomic approach to compare the roles of sample C and Neviotin A to decipher information on the activation of genes belonging to nine biological pathways, which play a pivotal role in triggering anticancer effects in MCF-7. Herein, we describe the anticancer potential of a chloroform extract of the marine sponge *Haliclona fascigera* (sample C) and Neviotin A in MCF-7 cells by measuring the (i) inhibition of cell proliferation and intracellular ROS production; (ii) cell cycle dysregulation and induction of apoptosis; (iii) transcriptome analysis using an array of 84 genes; (iv) translational activation of apoptotic proteins; and (v) the bioassay-guided fractionation of the extract and the identification of the most active compound.

## 2. Results

### 2.1. Cell Viability Analysis

**Figure 1:**
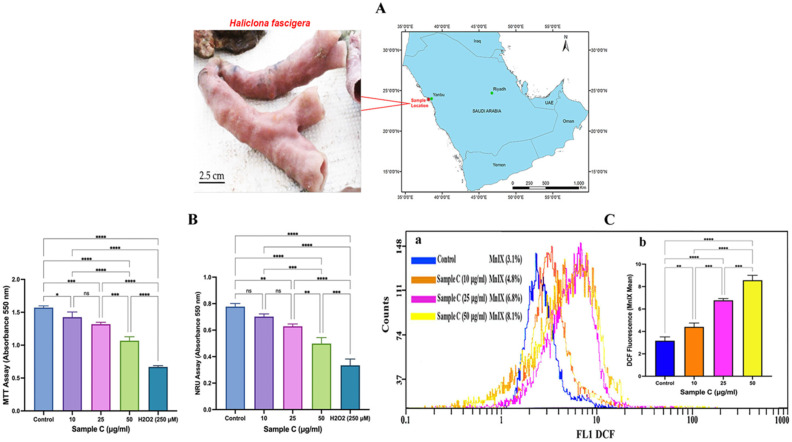
Chloroform extract (sample C) of a marine sponge (*Haliclona fascigera*) caused cytotoxicity and elevated the ROS level in MCF-7 cells. (**A**) Morphology and location details of *Haliclona fascigera* collected from the coastal area of the Red Sea, Yanbu, Saudi Arabia. (**B**) Effect of sample C on cell proliferation of MCF-7 cells after 24 h. Sample C induced a concentration-dependent reduction in MCF-7 survival analyzed using an MTT assay (* *p* = 0.0290, *** *p* = 0.0007, **** *p* < 0.0001, ns = nonsignificant) and NRU assay (* *p* = 0.0021, ** *p* = 0.0053, *** *p* = 0.0002, **** *p* < 0.0001, ns = nonsignificant). Each histogram is the mean ± SD of three experiments performed in triplicate wells. (**C**) (**a**) Representative flow cytometric peaks of DCF fluorescence recorded in sample C-treated MCF-7 cells show a greater shift on the logarithmic scale, indicating higher ROS generation in cells after 24 h of exposure. (**C**) (**b**) Each histogram in the inset is the mean ± SD of DCF fluorescence recorded in three experiments carried out in duplicate wells (** *p* = 0.0088, *** *p* = 0.0001, **** *p* < 0.0001). ANOVA one-way, post-hoc Tukey’s multiple comparisons test, MnIX: mean intensity.

*Haliclona fascigera*, collected from Yanbu, Red Sea, Saudi Arabia, was extracted with chloroform from the freeze-dried material to afford sample C. The obtained extract showed promising results in the MTT assay: after 24 h of exposure with varying concentrations (10, 25, and 50 μg/mL) of sample C, MCF-7 cell populations declined to 9.3%, 16.1%, and 25.1% (Figure 1B). The NRU assay also exhibited cytotoxicity in MCF-7 cells with increasing concentrations of sample C. After 24 h of incubation, 10, 25, and 50 μg/mL sample C induced 9.7%, 19%, and 29% reductions in the viability of cells, respectively (Figure 1B). Dimethyl sulfoxide (DMSO, 0.3%) as vehicle control showed no signs of cytotoxicity, and the cell population was found to be 98.5%.

### 2.2. Intracellular ROS generation

The representative flow cytometric peaks of sample C-treated MCF-7 cells exhibited a concentration-dependent shift in the DCF fluorescence on a logarithmic scale, which indicated strong ROS generation in cells (Figure 1C(a)). Quantitative analysis of sample C (10, 25, and 50 μg/mL)-treated cells exhibited significant 139%, 213%, and 270% increases in ROS over control (Figure 1C(b)).

### 2.3. Cell Cycle Deregulation by Sample C

**Figure 2:**
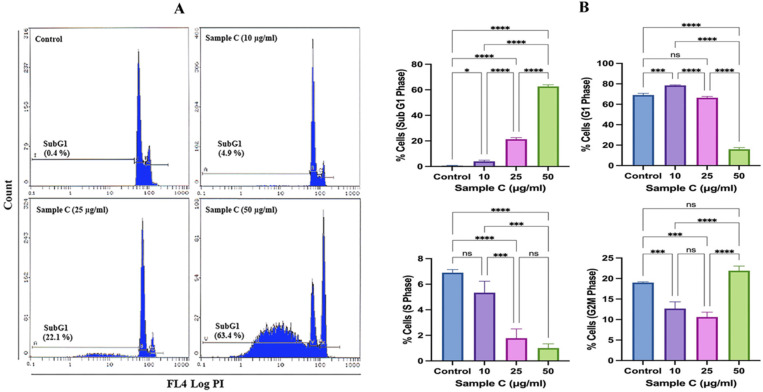

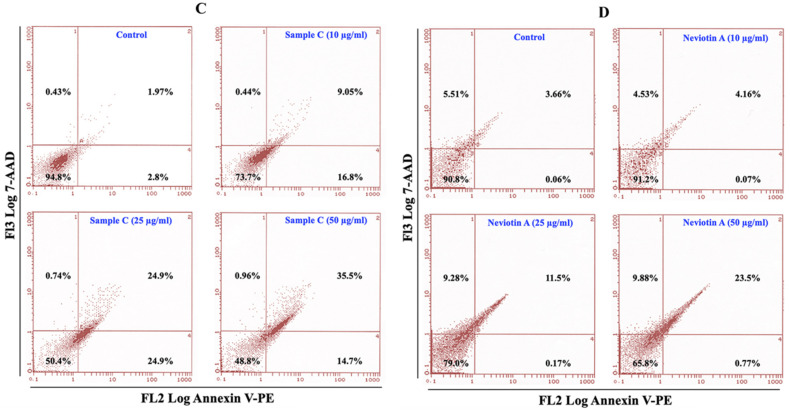
Sample C and Neviotin A (the most active compound, isolated and purified from sample C fractions 21–23, described in Section 2.4 below) triggered apoptotic cell death in MCF-7 cells. (**A**) Representative flow cytometric images of the MCF-7 cell cycle show an increase in the subG1 apoptotic peak after 24 h exposure with sample C. (**B**) Effect of sample C on the different phases of the MCF-7 cell cycle after 24 h of exposure to sample C. Each histogram represents the mean ± SD from three independent experiments. SubG1 (* *p* = 0.0150, **** *p* < 0.0001), G1 (*** *p* = 0.0001, **** *p* < 0.0001, ns = nonsignificant), S (*** *p* = 0.0005 and 0.0001, **** *p* < 0.0001, ns = nonsignificant), G2M (*** *p* = 0.0008 and 0.0001, **** *p* < 0.0001, ns = nonsignificant) (ANOVA one-way, post-hoc Tukey’s multiple comparisons test). G1, S, and G2M represent the percentages of cells in the normal phases of the cell cycle, and subG1 represents the percentage of cells that underwent apoptosis. (**C**,**D**) Bivariate flow cytometric analysis of sample C- and Neviotin A-treated MCF-7 cells. The dot plots in different panels exhibit the percent distribution of live (lower left quadrant), early apoptotic (lower right quadrant), late apoptotic (upper right quadrant), and necrotic (upper left quadrant) MCF-7 cells appeared after 24 h exposure to sample C and Neviotin A at increasing concentrations.

The representative cell cycle images exhibited concentration-dependent cell death in the sample C-treated MCF-7 cells (Figure 2A). The average data showed a significant increase in the apoptotic peak (subG1) at all tested concentrations. Compared to 0.61 ± 0.25% of background apoptotic cells in the control, sample C exposure of 10, 25, and 50 μg/mL showed significant 3.93 ± 0.96%, 21.26 ± 1.27%, and 62.6 ± 1.32% apoptotic cells (Figure 2B). The G1 and S phases gradually diminished with increasing concentrations. At the highest concentration of 50 μg/mL, a marginal but significant G2/M arrest was also observed, showing 21.9 ± 1.12% of cells in this phase. Flow cytometric analysis of apoptosis and necrosis in MCF-7 cells exposed to 50 μg/mL of sample C and Neviotin A (the most active compound, isolated and purified from sample C fractions 21–23, described in Section 2.4 below) exhibited 35.5% and 23.5% cells in the late apoptotic phase (upper right quadrant) (Figure 2C,D). Relatively necrotic cell death (upper left quadrant) in MCF-7 cells was not prominent after sample C and Neviotin A exposure (Figure 2C,D).

### 2.4. Bioassay-Guided Fractionation of Sample C

**Figure 3:**
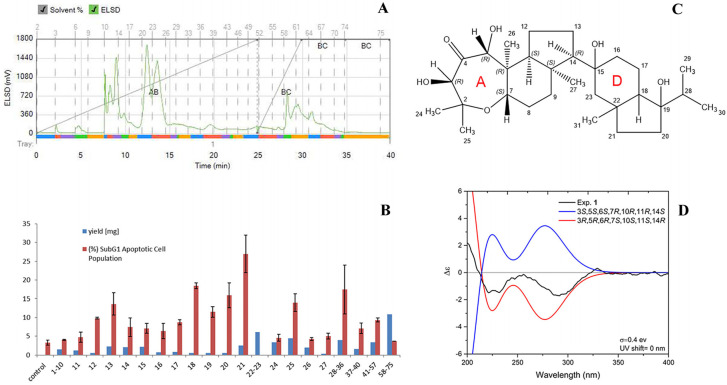
Bioassay-guided fractionation of sample C exhibited Neviotin A, causing greater cell death in MCF-7 cells. (**A**) ELSD chromatogram of the bioassay-guided fractionation of sample C. (**B**) Effect of the obtained fractions on MCF-7 apoptotic phase, analyzed in terms of subG1 peak in flow cytometry (50 μg/mL, *n* = 2, data shown as mean ± SD). (**C**) Chemical structure and absolute configuration of rings A/B/C of 3*R*, 5*R*, 6*R*, 7*S*, 10*S*, 11*S*, 14*R* Neviotin A. (**D**) Comparison of calculated (reduced molecule, rings D and E replaced by a methyl group) and experimental ECD spectra of Neviotin A.

In order to identify the active principal(s) of sample C, a part of the extract (52.5 mg) was subjected to silica gel column chromatography for bioassay-guided fractionation using a Reveleris X2 flash system (Büchi, Switzerland) with a gradient of *n*-hexane to ethyl acetate (0% to 100% in 25 min) to methanol (in 5 min to 100%; kept for 10 min). The eluate was monitored using an evaporative light scattering detector (ELSD) and collected according to peak detection into 75 test tubes (Figure 3A). The contents of tubes 1–10, as well as tubes 28–36, 37–40, 41–57, and 58–75, were combined to provide a total of 22 subfractions. Since tubes 21, 22, and 23 contained the major peak of the total ion chromatogram (TIC) of the liquid chromatography–mass spectrometry (LC-MS) analysis (ESI, negative modus) (Figure S3), fraction 21 was submitted for biological evaluation and fractions 22 and 23 were used for structure elucidation.

Biological evaluation of fractions 1–10, 11, 12, 13, 14, 15, 16, 17, 18, 19, 20, 21, 28–36, 37–40, 41–57, and 58–75 was achieved by measuring cell death (subG1) in MCF-7 cells using flow cytometry. MCF-7 cells in the subG1 phase (50 μg/mL) revealed not a single active principle but the presence of several active fractions like tubes 13, 18, or 25. A maximum of apoptotic cells with 27.0 ± 5.0% MCF-7 cells in the subG1 phase was found for fraction 21 (Figure 3B). LC-MS analysis of the combination of fractions 22 and 23 revealed a molecular weight of 506 deduced from the observed ions with *m*/*z* 551.6 [M + formate]^−^ and 1011.7 [2M-H]^−^. The recorded 1D- and 2D-nuclear magnetic resonance (NMR) spectra in CDCl_3_ and DMSO-*d*_6_ enabled the identification of the compound as Neviotin A [31,32] with an unclear relative configuration (Table S1 and Figures S4–S10). The nuclear Overhauser effect spectroscopy (NOESY) spectrum (measured in CDCl_3_) revealed a correlation from H-5 to OH-3 (δ_H_ 5.05 ppm; δ_H_ 3.19 ppm) displaying the ax-eq substitution of hydroxyl groups adjacent to the carbonyl group in ring A; from H-3 to CH_3_-24 (δ_H_ 1.21 ppm); from CH_3_-25 (δ_H_ 1.32 ppm) to H-7; and from H-7 to CH_3_-27 (δ_H_ 1.32 ppm). Considering that no NOE was observed from H-7 to CH_3_-26 (δ_H_ 0.68 ppm), substitution at ring A-B fusion was assigned as *trans*. Additional NOE from CH_3_-26 to H-11 and a lack of NOE from H-11 to CH_3_-27 and from CH_3_-27 to H-14 resulted in establishing the relative configuration of ring A/B/C as 3S*, 5S*, 6S*, 7R*, 10R*, 11R*, 14S*. Efforts to determine the relative configuration of rings D and E, including the use of a recorded rotating frame Overhauser effect spectroscopy (ROESY) spectrum and using another NMR-solvent (DMSO-*d*_6_), failed due to overlapping NMR signals and therefore left a remaining ambiguity of signal assignments. Nevertheless, to establish the absolute configuration of the molecule part consisting of three rings (A/B/C), electronic circular dichroism along with quantum chemical calculation was applied. To reduce the time of calculation, rings D and C were truncated and replaced by a methyl group, also considering the change of priority at C-10 and C-14. Conformational analysis and subsequent geometry optimization and minimization at DFT/wb97xd/6-31g(d,p)/CPCM/acetonitrile level resulted in two dominant conformers with populations of 52 and 48% (Figure S10). Further simulation of electronic circular dichroism (ECD) spectra at the TD-DFT/cam-b3lyp/6-31+g(d,p)/CPCM/acetonitrile level and its comparison with experimentally obtained ECD spectrum in acetonitrile revealed an opposite ECD spectrum, resulting in the unambiguous determination of the absolute configuration of the chiral carbons of ring A/B/C as 3*R*, 5*R*, 6*R*, 7*S*, 10*S*, 11*S*, 14*R*, as shown in Figure 3C,D.

### 2.5. Effect of Sample C and Neviotin A on the Apoptotic Pathway

**Figure 4:**
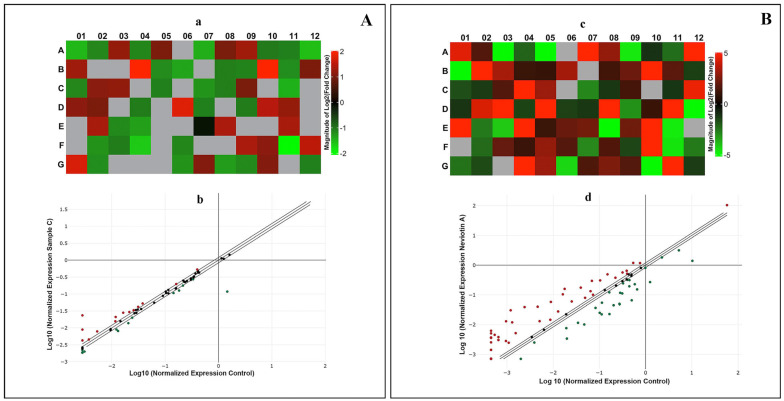
Sample C and Neviotin A caused transcriptomic changes in MCF-7 cells. qPCR array of human apoptosis signaling pathway genes in MCF-7 cells exposed to (**A**) sample C (25 μg/mL) and (**B**) Neviotin A (25 μg/mL) for 24 h. (**a**,**c**) Heat maps of all genes analyzed in MCF-7 cells exposed to sample C and Neviotin A. Red boxes are upregulated genes, and green boxes are downregulated genes. Expression of some genes was not detected in the analysis, shown as gray square boxes in the heat maps. (**b**,**d**) Scatterplots depicting the upregulation and downregulation of genes at the threshold value of >1.2-fold after exposure with sample C and Neviotin A. The list of genes upregulated and downregulated in the above analysis is listed in Supplementary Table S2.

MCF-7 cells exposed to sample C (25 μg/mL) for 24 h exhibited transcriptomic alterations in apoptosis pathways. RT^2^ profile PCR array data are expressed as the fold changes in expression obtained by comparing sample C-treated cells with untreated control. Among the array of 84 genes, a total of 14 genes were significantly upregulated in the sample C-exposed cells (Supplementary Table S2). Among the upregulated genes are *AKT1*, *BAX*, *BCL2L1*, *BCL2L2*, *BIRC5*, *CD70*, *CRADD*, *CYCS*, *DIABLO*, *IGF1R*, *LTBR*, *TNFRSF10B*, *TNFRSF1B*, and *TNFRSF21*. The 12 downregulated genes (fold change > 1.2) in sample C include *ABL1*, *BAG3*, *BCL2A1*, *BID*, *CASP2*, *CASP6*, *FADD*, *FAS*, *NOL3*, *NFKB1*, *TNFRSF1A*, and *TP73*. Heat maps of all upregulated and downregulated genes and scatterplots are shown in Figure 4A(a,b). 

Correspondingly, MCF-7 cells exposed to Neviotin A (25 μg/mL) for 24 h exhibited (fold change > 1.2) upregulation of 39 genes and downregulation of 27 genes (Supplementary Table S2). The upregulated genes were *ABL1*, *AIFM1*, *BAG3*, *BAK1*, *BCL2A1*, *BCL2L10*, *BCL2L11*, *BID*, *BIRC2*, *BIRC3*, *BIRC5*, *BIRC6*, *BRAF*, *CASP1*, *CASP10*, *CASP4*, *CASP7*, *CASP9*, *CD27*, *CD40LG*, *CIDEA*, *CYCS*, *DFFA*, *FAS*, *GAAD45A*, *HRK*, *IL10*, *LTA*, *NOD1*, *PYCARD*, *RIPK2*, *TNFRSF10A*, *TNFRSF11B*, *TNFSF10*, *TNFSF8*, *TP53BP2*, *TP73*, *TRADD*, and *TRAF3*. The genes downregulated by Neviotin A were *AKT1*, *APAF1*, *BAD*, *BAX*, *BCL2*, *BCL2L1*, *BNIP2*, *BNIP3*, *BNIP3L*, *CASP2*, *CD40*, *CD70*, *CIDEB*, *DAPK1*, *DIABLO*, *FADD*, *IGF1R*, *LTBR*, *NFKB1*, *TNF*, *TNFRSF10B*, *TNFRSF1A*, *TNFRSF1B*, *TNFRSF21*, *TNFRSF25*, *TRAF2*, and *XIAP*. The heat maps of all upregulated and downregulated genes and the scatterplot obtained from Neviotin A-exposed MCF-7 are shown in Figure 4B(c,d).

### 2.6. Translational and Transcriptional Activation of Apoptotic Proteins

**Figure 5:**
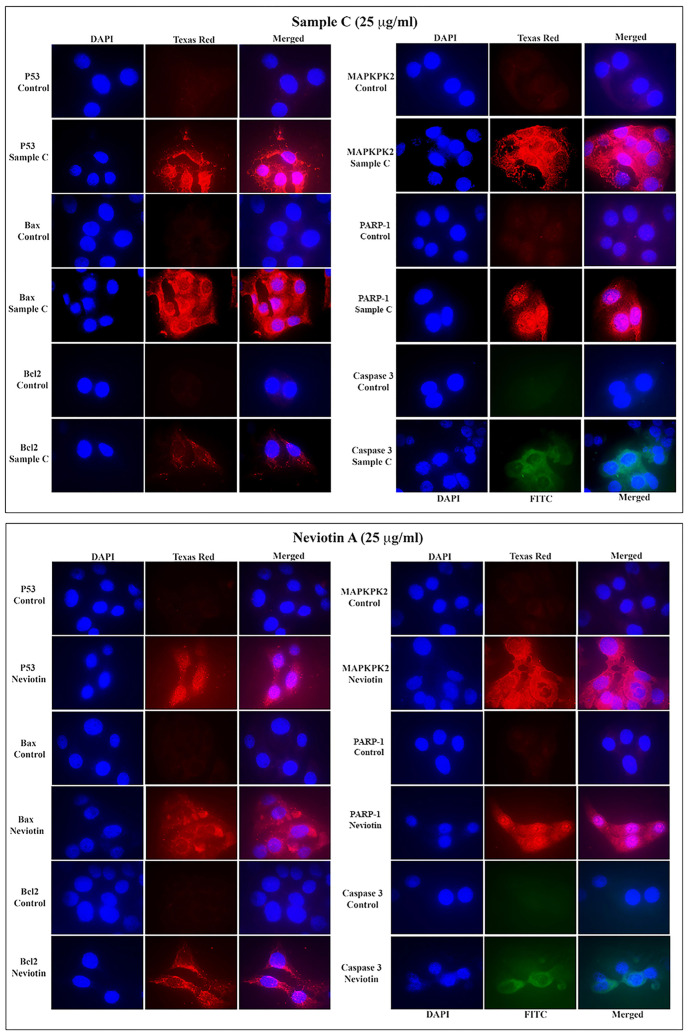
Sample C and Neviotin A activated the apoptotic proteins in MCF-7 cells. Immunofluorescence images showing the translational activation of apoptotic proteins in MCF-7 cells exposed to sample C (25 μg/mL) and Neviotin A (25 μg/mL) for 24 h. All images were captured using a high-resolution 1.5-megapixel CCD color camera (Nikon DS-Ri1, Japan). The fluorescence of DAPI and FITC was analyzed at the following excitation (340–380 nm) and emission (435–485 nm) bandwidths. The fluorescence of Texas Red was analyzed at excitation (540–580 nm) and emission (600–660 nm) bandwidths. Images were merged using Nikon image analysis software.

The fluorescent signal of apoptotic proteins (p53 and bax) in MCF-7 cells exposed to 25 µg/mL of sample C and Neviotin A was considerably high in the cytoplasm (Figure 5). Also, the fluorescence signals of MAPKPK2, caspase 3, and PARP-1 in sample C- and Neviotin A-treated cells showed cytoplasmic localization and the intensity of fluorescence was fairly consistent throughout a cell population. Remarkably, nucleolar localization of PARP-1 was observed in both sample C- and Neviotin A-treated cells (Figure 5).

**Figure 6:**
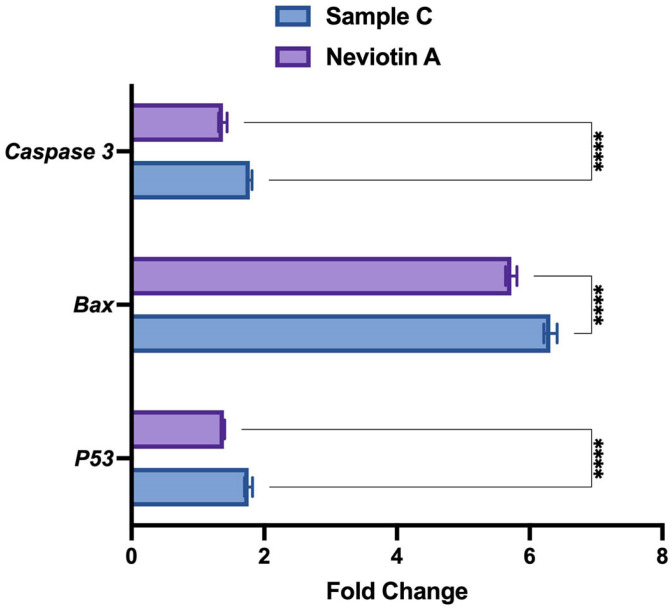
Sample C and Neviotin A changed the expression of apoptotic genes in MCF-7 cells. qPCR expression of some selected genes of apoptosis shows the upregulation of mRNA transcripts in MCF-7 cells exposed to sample C (25 μg/mL) and Neviotin A (25 μg/mL) for 24 h. **** *p* < 0.0001 (two-way ANOVA, Šídák’s multiple comparison test).

Quantification of *p53*, *bax*, and *caspase 3* genes using real-time PCR analysis also showed upregulation in sample C- and Neviotin A-treated cells. Compared to untreated control, MCF-7 exposed to 25 μg/mL of sample C showed 1.7-fold, 6.3-fold, and 1.7-fold upregulation of *p53*, *bax*, and *caspase 3* genes (Figure 6). Neviotin A (25 μg/mL)-exposed cells induced 1.4-fold, 5.7-fold, and 1.3-fold upregulation of *p53*, *bax*, and *caspase 3* genes, respectively.

## 3. Discussion

**Figure 7:**
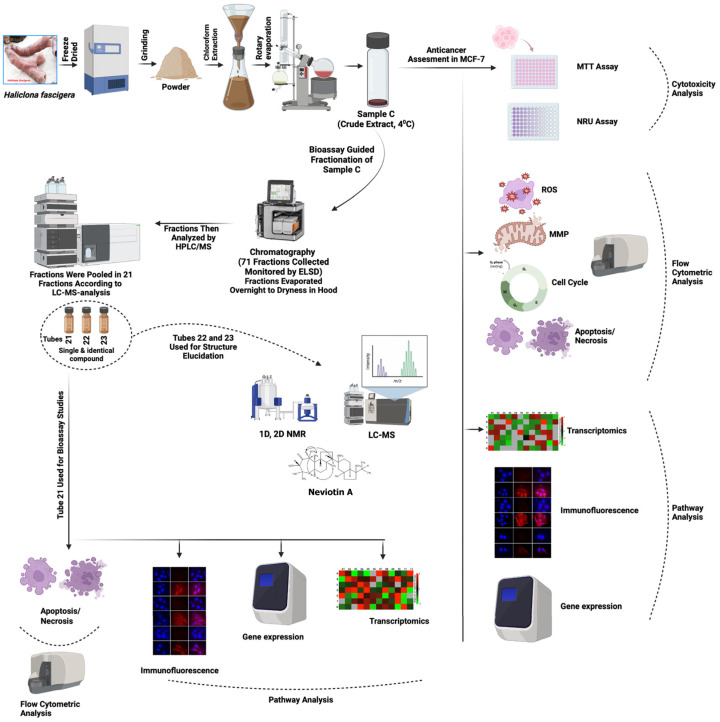
Flowchart of the working mechanism of sample C and Neviotin A. Illustration showing the overall working protocol implemented for the preparation of chloroform extract (sample C) from marine sponge (*Haliclona fascigera*) and bioassay-guided fractionation for the isolation, purification, and structure analysis of Neviotin A, the most active fraction. Different biological assays were performed to evaluate the anti-breast cancer potential of sample C and Neviotin A.

For several decades, marine natural products have been an invaluable source of therapeutic agents. The structural diversity of compounds isolated from marine organisms, especially sponges, serves as a privileged scaffold in the development of therapeutic agents [33]. Marine organism-derived anticancer drugs, *viz*., Ara-C and Halaven, specifically target the signaling intermediates in apoptosis-inducing pathways, known to modulate the process of carcinogenesis [34,35,36]. Extracts *Haliclona* sp. from North Carolina and Iran exhibited antibacterial and antifungal effects [37,38]. Bioassay-guided fractionation of *Haliclona* sp. extracts from Indonesia yielded papuamine and haliclonadiamine, which showed cytotoxicity and apoptosis in MCF-7, LNCap, Caco-2, and HCT-15 cells [39]. In spite of this, the anticancer effects of bioactive compounds isolated from the Red Sea *Haliclona* sp. is still obscure. The significance of our study is that it provides pathway-based information, especially from the perspective of mechanistic inhibition of breast cancer cells. In this line, we collected the marine sponge (*Haliclona fascigera*), prepared its chloroform extract (sample C), and evaluated its anti-breast cancer potency using MCF-7 cells that showed cytotoxicity, ROS generation, mitochondrial dysfunction, transcriptomic alterations, and cell death. Subsequently, we performed the bioassay-guided fractionation of sample C (Figure 7). Instead of an MTT-based data comparison, we have implemented a sensitive flow cytometric data comparison approach. In order to achieve this task, this study was planned to perform bioassay-guided fractionation to isolate bioactive compounds. In this correlation, we found the highest cell death in fraction 21 of the sample C extract and successfully isolated neviotine A from it. Consequently, we provided a broader picture of anticancer effects, starting with the crude extract (sample C) and ending with its most bioactive compound, i.e., Neviotin A.

In this study, the MCF-7 cell line was used due to its estrogen and progesterone receptor-positive properties. In addition, MCF-7, being a luminol A molecular subtype, is noninvasive, poorly aggressive, and has low metastatic potential [40,41,42,43]. These characteristics of MCF-7 cells have made them an appropriate cell line for breast cancer studies globally, including studies pertaining to anticancer drugs that have resulted in more practical knowledge for patient care relative to other breast cancer lines [40,44].

Cell proliferation endpoints (MTT and NRU) were achieved by initially exposing the cells to sample C. MTT and NRU exhibited a significant reduction in cell survival at all tested concentrations. Moreover, the NRU assay showed a higher effect on cell viability and indicated greater lysosomal damage. Cell survival data are in line with earlier studies, which have also demonstrated cytotoxicity by the extracts of *Haliclona* sp. [28,29,39]. Alterations in the delicate membranes of lysosomes may provoke their fragility, which affects the uptake and binding of neutral red dye, possibly by reducing the cellular lysosomal acid phosphatase activity [45]. Destabilization of lysosomes has been considered an earlier event in mitochondrial toxicity; simultaneously, intracellular ROS has been linked to aggravate such defects, causing the loss of mitochondrial membrane potential [46,47]. ROS at the physiological level effectively coordinates the regulation of lysosomes and mitochondria. While a higher ROS level is detrimental for cells owing to the reality that mitochondrial dysfunction damages the architecture of lysosomes [48,49]. Hence, we quantitated ROS generation in sample C-treated MCF-7 cells to ascertain its role as an oxidative stressor. Flow cytometric data showed an enhanced level of ROS generation at all concentrations, affirming that sample C encompasses active principles harboring ROS-generating capabilities to kill MCF-7 cells. The higher propensity for ROS generation is in accordance with a greater ROS level and oxidative stress in glioma cells exposed to the extract of a marine sponge (*Polymastia janeirensis*) [50]. Moreover, ROS act as a potent stimulus for the induction of apoptosis in cells. Especially when cells are under stress, ROS lead to cell death via the extrinsic or intrinsic pathway (mitochondrial) [51]. In this connection, ROS-induced killing of cancer cells mainly relies on oxidative stress-dependent apoptotic cell death [52].

Henceforth, we separately analyzed sample C and its silica gel chromatography fractions for apoptosis induction in MCF-7 cells. Due to the large number of fractions, it would not have been possible for us to compare 20 fractions with sample C in each experiment. Hence, we only evaluated the cell cycle analysis of all fractions and compared it with sample C. We found a concentration-dependent increase in the flow cytometric subG1 apoptotic peak in sample C as a whole. Also, a reasonable increase in the subG1 peak was recorded for its fractions, especially fraction 21, which provoked maximum apoptosis in the MCF-7 cells. The patterns of cell death indicated the possible role of apoptotic pathways, prompted by functional alterations of mitochondria and lysosomes [53,54]. It has been suggested that lysosomal membrane damage, as observed in the NRU assay, is known to release lysosome protease into the intracellular spaces, which affects adjacent cells and triggers cell death [55]. Sample C (50 µg/mL) treatment showed apoptosis (subG1) and G2/M arrest. Also, fraction 21 demonstrated higher apoptosis (subG1), which was confirmed as Neviotin A, specifying the fact that a common signal has been sent to prevent the damaged cells from further replication and to eliminate them [56]. It is known that cellular DNA repair mechanisms are highly conserved, and extensive DNA damage may lead to cell cycle arrest and cell death [57]. In these connections, bivariate flow cytometric analysis between sample C and Neviotin A was carried out to validate the activity of fraction 21, as well as to specify the type of cell death (i.e., apoptotic or necrotic). The annexin V-PE and 7-ADD data explicitly showed an early and late apoptotic effect in MCF-7, while the signal for necrosis was very weak after sample C and Neviotin A exposure. The annexin V^+^-PE cells in the lower right quadrant that appeared at all treated concentrations signified the breakage of the plasma membrane and externalization of phosphatidylserine (PS) due to the loss of membrane assembly, an indicator of early apoptosis. On the other hand, significant annexin V^+^–PE and 7-ADD^−^ cells in the upper right quadrant suggest the partial loss of cell membrane integrity in MCF-7 cells, eventually causing cell death due to late apoptosis but not necrosis [58]. Overall, cumulative analysis of bivariate data exhibits 51.16% cell death by sample C and 34.15% cell death by Neviotin A. These results are in agreement with the observed activity in other subfractions of sample C (Figure 3B), suggesting the presence of further active constituents in the chloroform extract. A possible justification for the greater cell death found in sample C could be due to the additive effects of other active fractions like 13, 18, or 25. Nonetheless, Neviotin A alone contributed substantially to the observed overall cell death when compared to sample C. Moreover, sample C and Neviotin A were analyzed for the activation of key apoptotic proteins. MCF-7 cells showed enhanced cytoplasmic localizations of p53, bax, bcl2, and MAPKPK2, which indicated the translational activation of apoptotic proteins as a consequence of sample C and Neviotin A exposure. During DNA damage, p53 is activated and translocated to the nucleus, where it can induce proapoptotic gene expression and accelerate the process of cell death [59]. Translational upregulation of p53 entails an impairment of cellular repair machinery, leading to the initiation of an apoptotic process to prevent the subsequent passage of mutations to other cells. On the other hand, mitochondrial toxicity observed in the MTT assay shows considerable importance as an early apoptotic event. The main intrinsic apoptotic pathway is characterized by mitochondrial dysfunction and activation of caspases [60]. The cytoplasm and nucleus of MCF-7 cells exhibited localization of PARP-1 after sample C and Neviotin A exposure, indicating its hyperactivation. PARP-1 is a nuclear protein attributed to participating in the DNA repair and protein modification processes through quick binding with the nicks and breaks in the damaged DNA. It is evidenced that higher levels of DNA damage in cells successively cause immense poly(ADP-ribosyl)ation by PARP-1, which leads to the activation of cell death [61]. Hence, it can be contemplated that sample C and Neviotin A exposure induced heavy DNA damage that was far beyond the repair capacity of MCF-7 cells to rescue them from cell death. On the other hand, MCF-7 cells exposed to sample C and Neviotin A showed caspase-3 localization in the cytoplasm. Caspase-3 plays a crucial role in intensifying apoptotic signaling via indirect or direct stimulation of downstream caspases [62]. Being a key player in orchestrating the apoptosis process, caspase-3 activation has been rendered a point of no return in the cascade of apoptotic signaling [63,64]. Relative to sample C, Neviotin A-treated MCF-7 also demonstrated a comparable effect by activating the apoptotic proteins, indicating its key role in executing the cell death process.

To unravel the role of sample C and Neviotin A in the induction of human apoptosis genes, we performed qPCR array experiments. The red and green boxes in the heat maps generated from sample C- and Neviotin A-treated MCF-7 cells exhibit the upregulation and downregulation of several genes. Although some genes were not expressed, they are shown as gray square boxes. The onset of apoptotic cell death in different cell types has been profoundly coupled with extrinsic and intrinsic pathways, a process in which impairment of mitochondrial functionality is deeply involved [65]. The extrinsic pathway is primarily instigated by the transmembrane death receptors belonging to the tumor necrosis factor (TNF) receptor gene superfamily (TNFRSF) [66]. Activation of key genes in TNFRSF is associated with the recruitment of FADD, dimerization of the death effector domain, and procaspase 8. The initiator caspase 8 stimulates caspase 3 to eventually trigger the extrinsic apoptotic pathway [66,67]. In the qPCR array, sample C- and Neviotin A-exposed MCF-7 cells showed upregulation of *TNFRSF10B*, *TNFRSF1B*, *TNFRSF21*, *TNFRSF10A*, *TNFRSF11B*, *TNFSF10*, *TNFSF8*, *LTBR*, and *TRAF*. The proteins encoded by these genes are members of the TNF superfamily. Moreover, copious amounts of non-receptor-mediated stimuli, including free radicals, DNA damage, and toxins, have been attributed to initiating the intrinsic apoptotic pathway [67]. The intrinsic pathway is principally governed by the members of the BCL-2 family, which is closely associated with the integrity of the mitochondrial outer membrane [67]. Similar to our data, *TNFRSF10B*, *TNFRSF1B*, and *TNFRSF21* genes were also upregulated in Jurkat E6-1 cells upon treatment with alkaloids extracted from *Tribulus terrestris* [68].

The damaged membrane leaks the apoptogenic factors (CYCS, DIABLO) into the cytoplasm, which, in association with others, activates the cascade of caspases to trigger apoptosis [69]. In this connection, sample C- and Neviotin A-exposed MCF-7 cells exhibited the upregulation of *CYCS*, *BCL2L1*, *BCL2L2*, *BCL2L10*, *BCL2L11*, *BCL2A1*, and *DIABLO* genes, thus affirming the inception of an intrinsic apoptotic pathway. Taken together, it can be speculated that sample C and Neviotin A trigger apoptosis in MCF-7 cells via both extrinsic and intrinsic pathways. These observations are parallel to the previous findings on the apoptotic gene expressions in in vitro and in vivo test models infected with HIV, which exhibited enhanced expression of genes pertaining to extrinsic, intrinsic, and executioner pathways [70,71].

Apart from the upregulation of the above TNF receptor family genes by sample C, Neviotin A exposure also upregulated *FAS*, *TRADD*, *RIPK2*, and *TRAF3* genes in MCF-7 cells. Ample evidence indicates that the upregulation of signal transducers has the ability to induce cell death due to their overexpression [72,73,74,75,76,77]. Together, FAS and TNFR1 have their conserved death domains in the cytoplasmic tails, which liaise with protein–protein interactions [78,79] and allow for the recruitment of other death domain-containing proteins, including FADD, TRADD, or RIP [72,73,74,75,76,77]. Comparatively, the TNFR2 cytoplasmic tail has exhibited its interaction with TRAF, leading to the activation of NFKB and the TNFR superfamily [76,80,81,82]. Hence, it can be hypothesized that Neviotin A-induced cell death in MCF-7 also encompasses additional signaling molecules using the FADD, TRADD, or RIP receptors. Furthermore, sample C-exposed cells exhibited upregulation of *BIRC5*. Correspondingly, Neviotin A-treated cells upregulated *BIRC2*, *BIRC3*, *BIRC5*, and *BIRC6* genes. These genes are members of the inhibitors of apoptosis (IAPs) family. *BIRC5*, unlike other IAPs, has functions to regulate mitosis and inhibit cell death. Consequently, its marked upregulation correlates with tumor resistance to chemotherapy [83,84]; hence, the *BIRC5* gene is a promising target for anticancer interventions [85,86,87]. The upregulation of *BIRC* family genes in MCF-7 cells clearly suggests that the inhibition threshold to control apoptosis was surpassed, eventually causing cell death. MCF-7 showed the upregulation and downregulation of fewer genes after sample C exposure. Relatively, MCF-7 cells treated with the purified and most active principle (i.e., Neviotin A) displayed stimulation of numerous genes. Therefore, the strong anticancer property of sample C can be attributed to Neviotin A. Such variations may be related to the coexistence of several active constituents in sample C, which may have interfered with the functionality of other genes. However, Neviotin A exposure presented a wider magnitude of activated genes, whose functions were unmasked in the cells. It can be summarized that the apoptotic potential of sample C and Neviotin A was far beyond the capacity of MCF-7 cells to rescue them from cell death. A previous study on Neviotin A isolated from the marine sponge *Siphonochalina siphonella* also demonstrated its antiproliferative effects in cancer cell lines, including MCF-7 [32].

We performed bioinformatic analysis to identify the miRNA regulators in MCF-7 cells targeting the upregulated and downregulated genes after sample C and Neviotin A exposure. The miRNA-gene interaction map of sample C-treated cells showed 326 miRNAs were involved in the upregulation of genes (Figure S11A, left panel; Supplementary Table S3), while 164 miRNAs targeted the downregulated genes in MCF-7 exposed to sample C (Figure S11A, right panel; Supplementary Table S3). In the case of Neviotin A, 260 miRNAs targeted the upregulated genes in cells (Figure S11B, left panel; Supplementary Table S4). A total of 509 miRNAs were involved in the downregulation of genes in MCF-7 cells exposed to Neviotin A (Figure S11B, right panel; Supplementary Table S4). The role of miRNA can be understood by the fact that a single miRNA can control several target genes. On the other hand, multiple miRNAs can control a single target gene. Similar to our finding, an environmental pollutant (zearalenone)-exposed Leydig cell line (TM3) also demonstrated the potential connection of the miRNA–gene network under toxicological repercussions in exposed cells [88]. Hence, it can be argued that the interdependency of the miRNA–gene network plays a crucial role in the functional operation of allied pathways. Our bioinformatic data provide a theoretical basis for this assumption. Hence, the apoptotic pathway in MCF-7 after sample C and Neviotin A exposure is not only restricted to gene regulation; a variety of miRNA-regulated networks are also involved, which are deemed to have been thoroughly investigated.

## 4. Materials and Methods

### 4.1. General

High-performance liquid chromatography with photodiode array detection (HPLC-DAD) analysis was conducted on an Agilent HP1100 system (Waldbronn, Germany) equipped with an autosampler, DAD, and column thermostat. For high-performance liquid chromatography with electrospray mass spectrometry (HPLC-ESIMS) experiments, the instrument was connected to an Esquire 3000 plus (Bruker Daltonics, Bremen, Germany) ion trap using electrospray ionization (ESI). LC parameters: stationary phase: Phenomenex MAX-RP 5 μm, 4.6 × 150 mm; mobile phase: A = H_2_O + 0.9% formic acid + 0.1% acetic acid, B = acetonitrile + 0.9% formic acid + 0.1% acetic acid; gradient: 0 min: 2% B; 30 min: 98% B; 45 min: stop; temp.: 30 °C, flow: 1 mL/min inj. vol.: 10 μL, sample conc.: extract 5 mg/mL; fractions 1 mg/mL in MeOH. MS parameter: split, 1:5; ESI, negative mode; spray voltage, 4.5 kV; dry temperature, 325 °C; drying gas flow rate, 8.00 L/min; nebulizer gas, 30 psi; mode, scan range: *m*/*z* 100–1200. One- and two-dimensional NMR experiments were recorded on a Bruker Avance II 600 spectrometer (Bruker) operating at 600.19 MHz (^1^H) and 150.91 MHz (^13^C) at 300 K (chemical shifts δ in ppm, coupling constants *J* in Hz), with deuterated chloroform (CDCl_3_ or DMSO-*d*_6_) as solvent.

### 4.2. Collection of Sponge

Sponge (*Haliclona fascigera*) was collected from the Red Sea coral reefs at a depth of 12 m around 7 km offshore (N23° 54.682, E38° 09.130) from Yanbu Industrial Park, Yanbu, Saudi Arabia (Figure 1A). The sponge was identified as *Haliclona fascigera* by the Environmental Control Department, Royal Commission for Jubail and Yanbu, Saudi Arabia. All samples were kept frozen at −20 °C until extraction.

### 4.3. Extract Preparation and Activity-Guided Isolation

The freeze-dried sample C (2.0 g) was powdered, extracted with chloroform (3 × 50 mL) each time for 24 h at room temperature, and filtered through Whatman No. 1 filter paper. Obtained filtrates were combined and evaporated to complete dryness under reduced pressure by using Büchi Rotavapor (Model R-215) at 40 °C, yielding 386 mg of crude extract named sample C. The dried sample C was redissolved in DMSO and used for evaluating its cytotoxicity against MCF-7 cells (a detailed method is presented below). The preliminary screening of the extract showed significant activity, which prompted activity-guided isolation of the active principal(s). Therefore, a small amount of the chloroform extract (52.5 mg) was suspended in 3 mL of *n*-hexane and mixed with 500 mg of silica gel (40–60 µm). The dried sample was transferred into a 3 g solid loader of a Reveleris X2 flash system (Büchi, Switzerland) and fractionated over a 4 g HP silica column (20 µm) using a gradient of *n*-hexane to ethyl acetate (0% to 100% in 25 min) to methanol (in 5 min to 100%; kept for 10 min). The applied flow rate was set to 5 mL/min. The eluate was monitored by ELSD (setting: high sensitivity, threshold 20 mV, high slope detection) and automatedly collected according to peak detection into 75 test tubes. The fractions were evaporated to dryness overnight at room temperature in a hood and analyzed using HPLC/MS. The fractions were pooled according to the results of the LC MS analysis into 21 fractions. The analysis of tubes 21, 22, and 23 showed the presence of a single and identical compound. Therefore, tube 21 was used for bioassay studies, while tubes 22 and 23 were combined (6.12 mg) and subjected to further analysis (1D-, 2D- NMR, and LC-MS) for structure elucidation, which enabled the identification of the compound as Neviotin A, with an unclear stereochemistry. In order to simulate ECD spectra, the 3D structure of the relevant part of the isolated compound (described in Figure 3) was subjected to Schrodinger MacroModel 9.1 (Schrodinger, LLC, New York, NY, USA) to perform conformational analysis using OPLS-3 force filed in the gas phase. The number of steps was set high enough to include all the important conformers. The conformers obtained (seven conformers in total) at the energy window of 5 Kcal·mol^−1^ optimized at the DFT/wb97xd/6-31g(d,p)/CPCM/acetonitrile level of theory using Gaussian 16. Vibrational frequencies were calculated at the same level, and no imaginary frequencies were observed. In total, two dominant conformers were obtained with populations of 52 and 48% (Figure S1). UV and ECD simulations were performed by implementing TD-DFT/cam-b3lyp/6-31+g(d,p)/CPCM/acetonitrile, and the curves obtained were extracted by SpecDis v.1.7 software with a half-band of 0.4 eV. No UV correction was required. The spectrum obtained was compared to the experimental spectrum recorded in acetonitrile with the Jasco J-1500 instrument (Easton, MD, USA).

### 4.4. Cell Culture and Cell Proliferation Assays

Sample C-induced proliferation inhibition of MCF-7 cells was analyzed using MTT and NRU assays. The rationale for the selection of concentrations was based on a wide screening of proliferation inhibition in MCF-7 cells, ranging from low to high (10, 25, 50, 100, 250, 500, and 1000 μg/mL) concentrations of *Haliclona fascigera* extracted with ethanol, chloroform, or water. Based on the highest proliferation inhibition by the chloroform extract (sample C) in MTT and NRU assays, the following sublethal concentrations (10, 25, and 50 μg/mL) were selected for further experiments (Figure S2). Hydrogen peroxide (H_2_O_2_) (250 μM), as a positive control, was used to separately expose MCF-7 cells for 24 h at 37 °C in a CO_2_ (5%) incubator. As a negative control, MCF-7 cells were treated separately with DMSO (0.3%) present in the highest concentration of sample C (50 μg/mL) under identical conditions [58]. On the other hand, MCF-7 cells were grown for 24 h at 37 °C in a CO_2_ (5%) incubator with 10, 25, and 50 μg/mL of sample C in a 96-well plate. After exposure, MTT dye (5 mg/mL) was added to each well and incubated for 4 h at 37 °C, followed by absorbance measurement at 550 nm on a microplate reader (Multiskan Ex, Thermo Scientific, Vantaa, Finland). The NRU assay was carried out by adding neutral red dye (50 μg/mL) to each well, followed by incubation for 3 h. Cells were washed off rapidly with a solution containing 1% calcium chloride and 0.5% formaldehyde. A mixture of acetic acid (1%) and ethanol (50%) was added to each well and incubated for 20 min at 37 °C. The aqueous medium was carefully aspirated, and 200 µL of DMSO was added to each well, followed by an absorbance measurement at 550 nm [58].

### 4.5. Flow Cytometric Quantitation of ROS

MCF-7 cells exposed to 10, 25, and 50 μg/mL of sample C for 24 h were detached using trypsin EDTA (0.25%). Cells were spun down at 3000 rpm for 3 min and washed twice with cold phosphate buffer saline (PBS), followed by the resuspension of cells in 500 µL of Ca^2+^ and Mg^2+^-free PBS containing 5 µM of fluorescent dye (DCFH-DA) dye. All cells were incubated for 60 min at 37 °C in the dark, followed by washing with PBS. The fluorescence of DCF was recorded upon excitation at 488 nm in the FL1 Log channel through a 525 nm bandpass filter on a Beckman Coulter flow cytometer (Coulter Epics XL/Xl-MCL, Beckman Coulter, Inc., Brea, CA, USA) [89].

### 4.6. Cell Cycle Dysregulation

Sample C treated (10, 25, and 50 μg/mL) MCF-7 cells were trypsinized and centrifuged at 3000 rpm for 5 min. Ice-cold 70% ethanol (500 μL) was added to fix the cells in each tube and left for 1 h at 4 °C. Cells were spun down at 3000 rpm for 5 min and washed twice with PBS. Cell pellets were suspended in PBS containing 0.1% Triton X-100, 50 µg propidium iodide (PI)/mL, and 50 μg/mL RNase A for 1 h at 30 °C in the dark. Propidium iodide (PI) fluorescence from 10,000 events was acquired on a flow cytometer through the FL4 Log channel via a 675 nm bandpass filter. The data were analyzed using Coulter Epics XL/XL-MCL, System II Software, Version 3.0. Cell cycle deregulation with all fractions (1–10, 11, 12, 13, 14, 15, 16, 17, 18, 19, 20, 21, 24, 25, 26, 27, 28–36, 37–40, 41–57, 58–75) from sample C was separately analyzed by following the above method [89].

### 4.7. Apoptosis and Necrosis Analysis

MCF-7 cells were separately treated with sample C (10, 25, and 50 μg/mL) and Neviotin A (isolated and purified from sample C) (10, 25, and 50 μg/mL) for 24 h at 37 °C. Post-exposure cells were harvested and processed according to the manufacturer’s protocol (Annexin V Apoptosis Kit PE # 736518, Beckman Coulter, Inc., Brea, CA, USA). Briefly, cells were washed twice with ice-cold PBS and pelleted at 3000 rpm for 4 min. Pellets were resuspended at a density of 1 × 10^6^ cells/mL in 1× binding buffer. Cell suspension (100 µL) from each concentration was separately incubated on ice for 15 min with annexin V-PE (10 μL) in the dark, followed by the addition of 7-AAD (10 μL) and 1× binding buffer (380 μL) in all tubes. Cells were kept on ice and analyzed within 30 min after staining. Fluorescence from 10,000 events was recorded on the flow cytometer through channels FL2 Log AnnexinV-PE (570 nm) and FL3 Log 7-AAD (585 nm) [58].

### 4.8. Transcriptome Analysis by qPCR Array

Sample C and Neviotin A induced transcriptional changes in the human apoptosis genes, which were analyzed using a qPCR array. In brief, RNA was isolated from sample C (25 μg/mL)- and Neviotin A (25 μg/mL)-exposed MCF-7 cells using a commercially available kit (RNeasy Mini Kit, Cat. No. 74106, Qiagen, Hilden, Germany). Purification of RNA was performed using the PureLink™ RNA Mini kit (Thermo Fisher Scientific, Waltham, MA, USA) by Invitrogen^®^ automated system, and its purity was verified on a Nanodrop 8000 spectrophotometer (Thermo Fisher Scientific, Waltham, MA, USA). cDNA synthesis was performed using 1 μg of total RNA by the RT^2^ First Strand Kit (Cat. No. 33040, Qiagen, Germantown, MD, USA). Expressional changes in the 84 genes responsible for human apoptosis were quantified using the 96-well format of the RT^2^ Profiler™ PCR Array (Cat. No. PAHS-012F-24, SABiosciences Corporation, Frederick, MD, USA). cDNA equivalent to 1 μg of total RNA was used for each array. The array plates were run on Roche^®^ LightCycler^®^ 480 (Roche Diagnostics, Rotkreuz, Switzerland) following the recommended cycling programs. Online software from SABiosciences Corporation, Frederick, MD, was used to analyze the expression data. Sample C and Neviotin A expression results were normalized to the average Ct values of five housekeeping genes (*ACTB*, *B2M*, *GAPDH*, *HPRT1*, and *RPLP0*) expressed with respect to the untreated control. RT-PCR array data were evaluated from at least three independent experiments, and the resultant ^2−ΔΔ^Ct values were combined to calculate the average fold regulation values. Online bioinformatic tools from Qiagen (Gene Globe) were used for developing the fold change scatter plots, heat maps, and miRNA network [89,90].

### 4.9. Immunofluorescence Analysis

MCF-7 cells at a density of 1 × 10^4^ cells/well were independently exposed to sample C (25 µg/mL) and Neviotin A (25 µg/mL) in 16-well Nunc glass chamber slides for 24 h. The media were discarded, and cells were fixed with 70% chilled methanol at −20 °C for 20 min, followed by washing with PBS. Cells were further incubated with 5% BSA for 1 h, followed by washing with PBS. Primary antibodies anti-p53 (Cat. no. sc-6243), anti-bax (Cat. no. sc-526), anti-bcl2 (Cat. no. sc-783), anti-MAPKPK2 (Cat. no. sc-7871), anti-PARP-1 (Cat. no. sc-25780), anti-caspase 3 (Cat. no. sc-7148) (Santa Cruz Biotechnology Inc., Santa Cruz, CA, USA) at the recommended dilution of 1:50 were added in separate wells of control and treated cells and allowed to incubate for 1 h at room temperature. After the incubation, all primary antibodies were discarded, and cells were washed five times with a mixture of 1% BSA and 0.001% Triton-X solution. Cells were then incubated with the recommended dilution (1:100) of secondary antibody (goat anti-rabbit IgG-TR Cat. No. sc-2780). In the case of caspase 3, cells were incubated with a 1:100 dilution of goat anti-rabbit IgG-FITC (Cat. No. sc-2012) (Santa Cruz Biotechnology Inc., USA) for 1 h at room temperature in the dark, followed by a thorough washing of cells with PBS. Nuclear staining was carried out by adding 300 nM of 4′,6-diamidino-2-phenylindole (DAPI) for 5 min, followed by washing with PBS. Cells were then overlaid with Prolong Gold Antifade Mountant (Cat. No. P36930, Thermo Fisher, Carlsbad, CA, USA) and covered with a coverslip. The immunofluorescence of proteins was captured by the use of a fluorescence microscope (Nikon Eclipse 80i, Minato-ku, Tokyo, Japan) [91].

### 4.10. Transcriptional Upregulation of Apoptotic Genes

Real-time PCR expression of some selected genes (*p53*, *bax*, and *caspase 3*) from sample C (25 µg/mL)- and Neviotin A (25 µg/mL)-treated MCF-7 cells was carried out to validate the immunofluorescence data [89]. In brief, RNA isolation, purification, and quantitation were performed as described above. The first-strand cDNA synthesis was performed using 1 μg of total RNA and 100 ng of oligop (dT)12–18 primer and MLV reverse transcriptase (GE Health Care, UK) following the manufacturer’s protocol. The following set of primers were used for *p53* (5′ F-CCCAGCCAAAGAAGAAACCA-3′, 5′ R-TTCCAAGGCCTCATTCAGCT-3′), *bax* (5′ F-TGCTTCAGGGTTTCATCCAG-3′, 5′ R-GGCGGCAATCATCCTCTG-3′), *caspase 3* (5′ F-ACATGGCGTGTCATAAAATACC-3′, 5′ R-CACAAAGCGACTGGATGAAC-3′), *HPRT* (5′ F-CCACTCCTCCACCTTTGAC-3′, 5′ R-ACCCTGTTGCTGTAGCCA-3′). Normalization of the gene expression was performed using *HPRT* as an internal housekeeping gene. LightCycler^®^ 480 was used to measure the fluorescence of SYBR Green I Master (Cat. No. 04707516001, Roche Diagnostics, Switzerland). A typical PCR mixture (20 μL) contained 10 μL of qPCR GreenMaster, 2.5 μL of 100 ng of cDNA, and 7.5 pmol of each primer. Instrument conditions were kept as follows: initial heat-denaturing step at 95 °C (ramp rate 4.4 °C/s) for 10 min; 45 cycles at 95 °C for 20 s (ramp rate 4.4 °C/s); annealing at 58 °C for all the primers for 20 s (ramp rate 2.2 °C/s); and product elongation and signal acquisition (single mode) at 72 °C for 20 s (ramp rate 4.4 °C/s). A template for negative control was used as water, and amplifications were included with each PCR run. Fold changes in the gene were analyzed using the ^2−ΔΔ^Ct method.

### 4.11. Statistical Analysis

Statistical significance was determined using GraphPad Prism 9 by performing normality (Shapiro–Wilk) and equal variance (Brown–Forsythe) tests with a subsequent ANOVA (one-way) post-hoc (Tukey’s multiple comparisons) test at *p* < 0.05. ANOVA (two-way) was performed using Šídák’s multiple comparisons test.

## 5. Conclusions

We found that *Haliclona fascigera* extract (sample C) at low concentrations has the potential to induce transcriptomic alterations and cell death in MCF-7 cells. The MTT and NRU data clearly demonstrated that sample C provoked cytotoxicity after 24 h of exposure. The treated cells exhibited oxidative stress, manifested as ROS generation. Flow cytometric data elucidated that sample C can trigger cell death exclusively by apoptosis but not by necrosis. The highest percentage of cell death induced by the bioactive fraction (21) of sample C, identified as Neviotin A, can be attributed to possessing anticancer properties. However, components other than Neviotin A may also play a synergistic role in triggering cell death in MCF-7 cells, which warrants future studies. Transcriptome analysis revealed the crosstalk between different apoptotic pathways, especially the genes encoding functions in apoptosis and cell death. We provide novel information on the fact that *Haliclona fascigera* extract exhibits anticancer properties against breast cancer via the activation of *BIRC5*, *AKT1*, MAPK, and *TNFRSF1A* genes. Neviotin A from *Haliclona fascigera* extract could be utilized for the future development of a chemotherapy drug for breast cancer. Nonetheless, to understand the related biological meaning, translational analysis and antitumor studies of Neviotin A are warranted in suitable in vitro and in vivo test models.

## Data Availability

All data are provided in this manuscript.

## Supplementary Materials

The following supporting information can be downloaded at: https://www.mdpi.com/article/10.3390/molecules28176289/s1, Figure S1: Population of the conformers obtained at wb97xd/6-31g(d,p)/CPCM/acetonitrile. Figure S2: Screening of *Haliclona fascigera* extracts on cell proliferation of MCF-7 cells after 24 h. Figure S3; TIC of the LC-MS analysis (ESI, negative modus) of sample C (5 mg/mL) and selected subfractions of sample C (each 1 mg/mL). Figure S4; ^1^H-NMR-spectrum in CDCl_3_. Figure S5; ^13^Carbon NMR spectrum in CDCl_3_. Figure S6; COSY-spectrum in CDCl_3_. Figure S7; HSQC-spectrum in CDCl_3_. Figure S8; HMBC-spectrum in CDCl_3_. Figure S9; NOESY-spectrum in CDCl_3_. Figure S10; TOCSY-spectrum in CDCl_3_.Table S1: NMR data of Neviotin A measured in CDCl_3_ or DMSO-*d*_6_. Figure S11; miRNAs-gene network map. Table S2: List of genes up- and downregulated in qPCR array. Tables S3 and S4: Number of miRNAs targeting an individual gene in sample C and Neviotin A-exposed MCF-7 cells.
