# Supplementary material for: Marine Natural Compound (Neviotin A) Displays Anticancer Efficacy by Triggering Transcriptomic Alterations and Cell Death in MCF-7 Cells
Source: Molecules. 2023 Aug 28;28(17):6289. doi: 10.3390/molecules28176289 (PMC10488820; doi:10.3390/molecules28176289)
Supplement: Supplementary file 1 [file molecules-28-06289-s001.zip › molecules-2455250-supplementary.pdf]

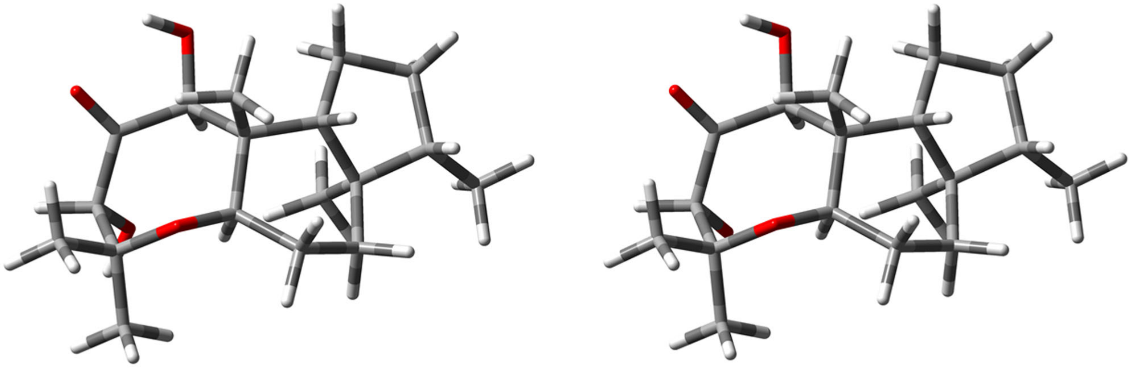

C1: 52%   C2: 48%

Figure S1. Two dominant conformers of Neviotin A obtained with population of 52 and 48%. Population of the conformers obtained at wb97xd/6-31g(d,p)/CPCM/ acetonitrile.

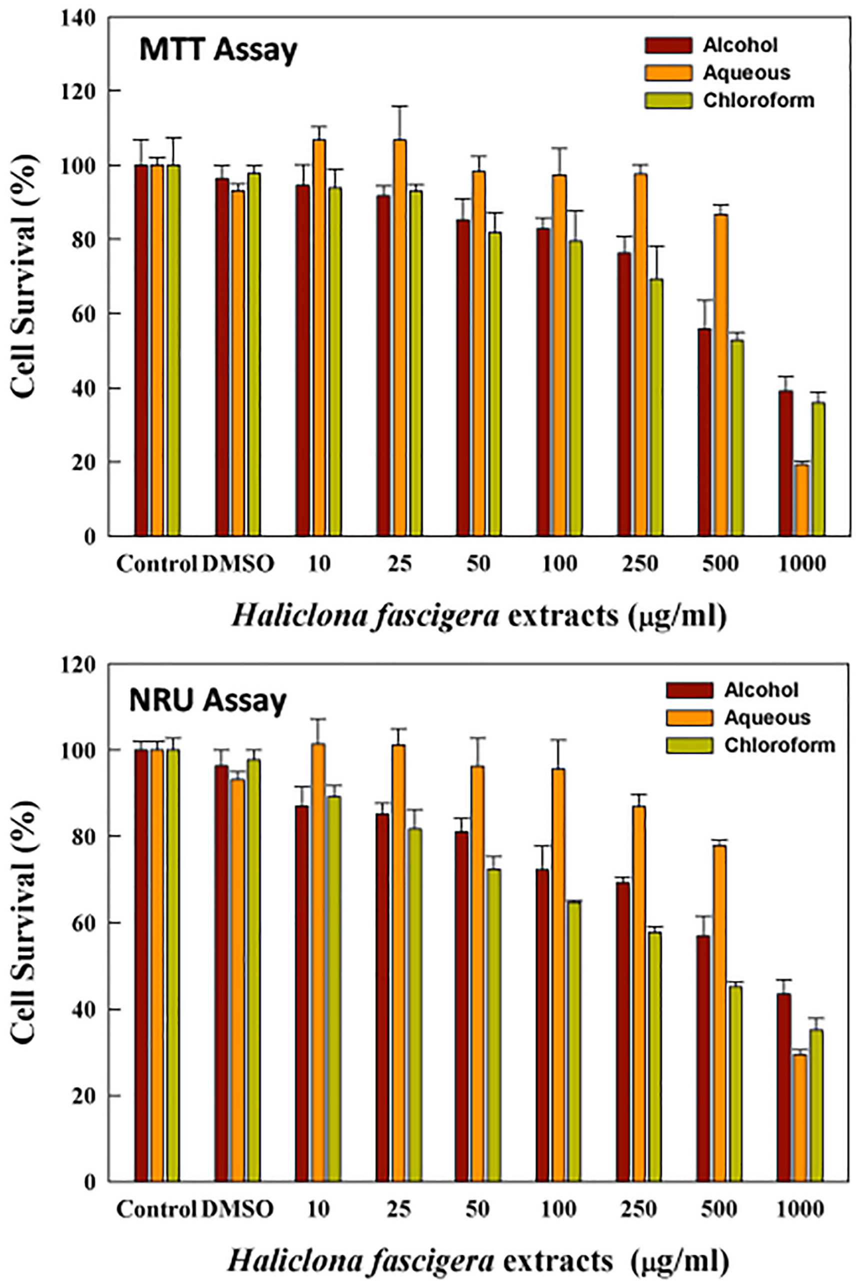

Figure S2. *Haliclona fascigera* extracts declined the survival of MCF-7. Screening of *Haliclona fascigera* extracts on cell proliferation of MCF-7 cells after 24h. Different extracts ethanol (alcohol), water (aqueous), chloroform (sample C) showed varying level of cell survival reduction in MCF-7 analyzed by MTT and NRU assays. Each histogram is the mean ± SD of three experiments done in triplicate wells.

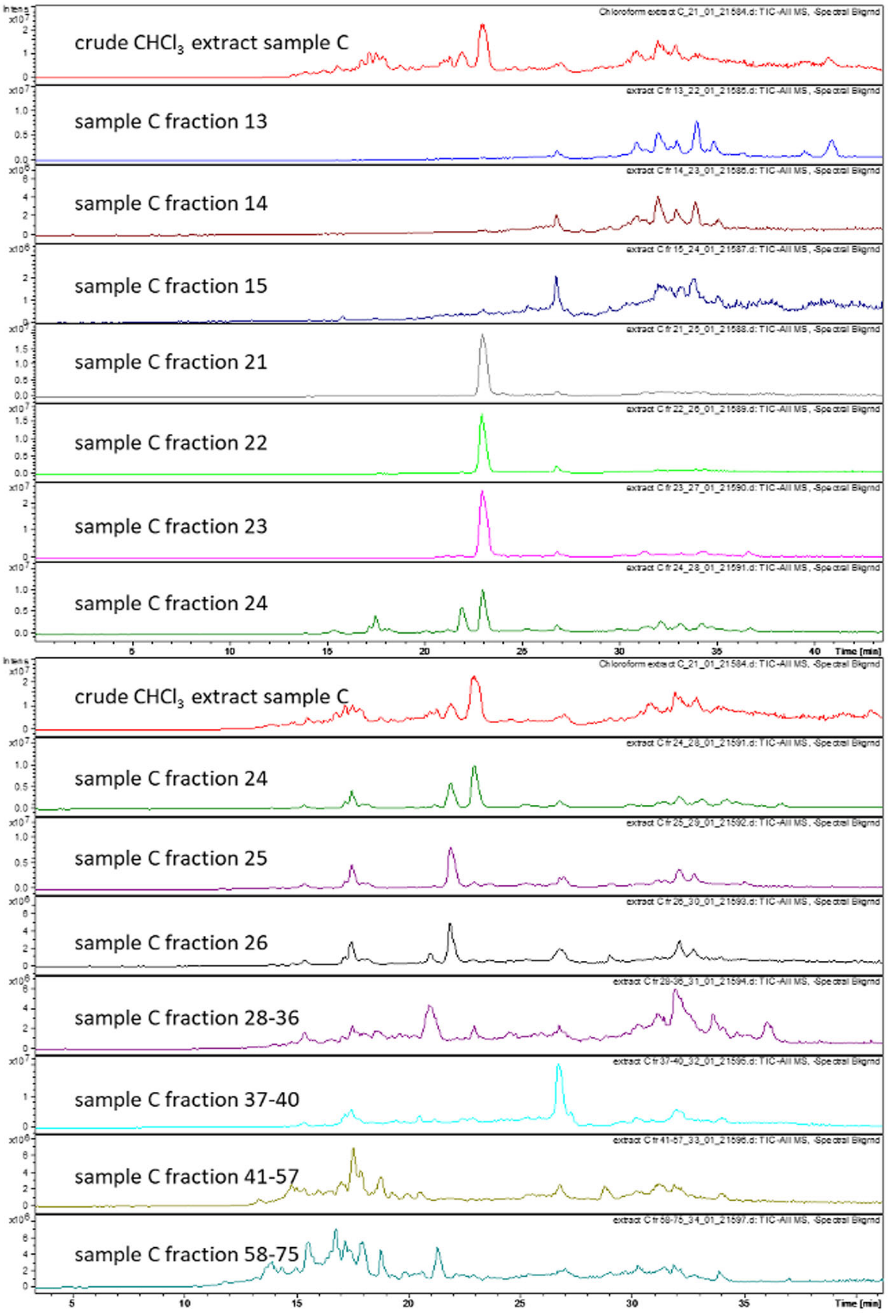

Figure S3. Bioassay guided fractionation of sample C. TIC of the LC-MS analysis (ESI, negative modus) of sample C (5 mg/ml) and selected subfractions of sample C (each 1 mg/ml). For analytical conditions, see section 4.3.

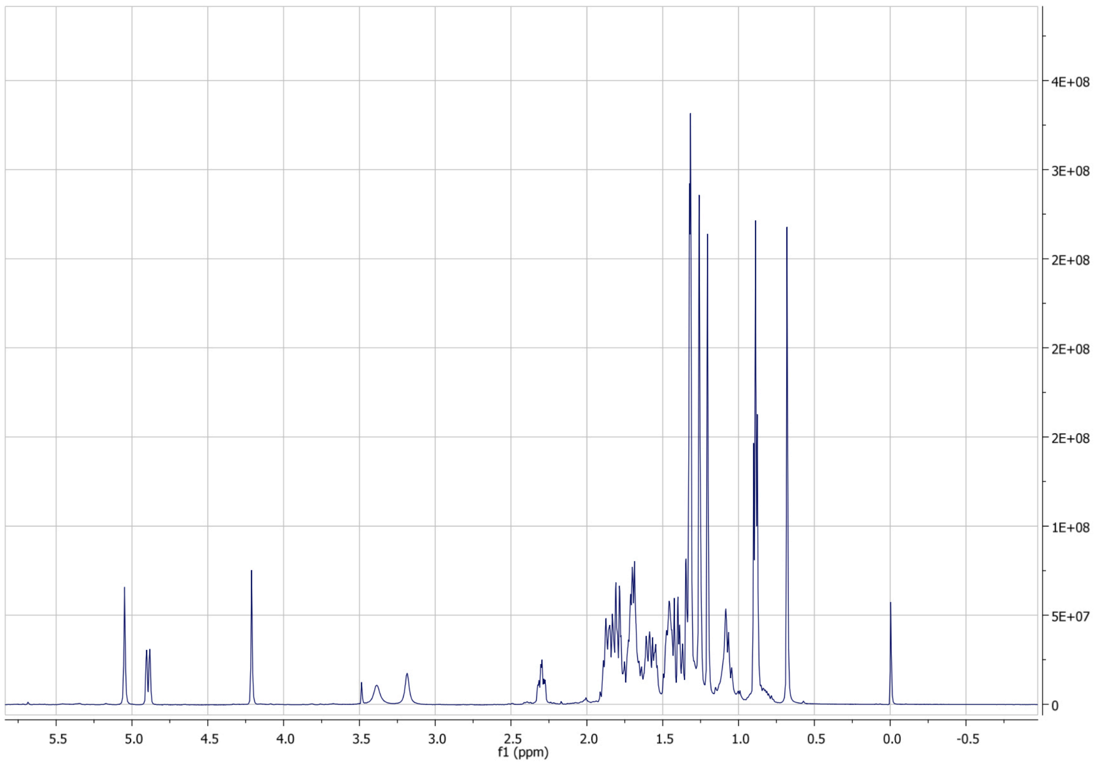

Figure S4. ^{1}H-NMR of Neviotin A. ^{1}H-NMR-spectrum measured in $CDCl_3$.

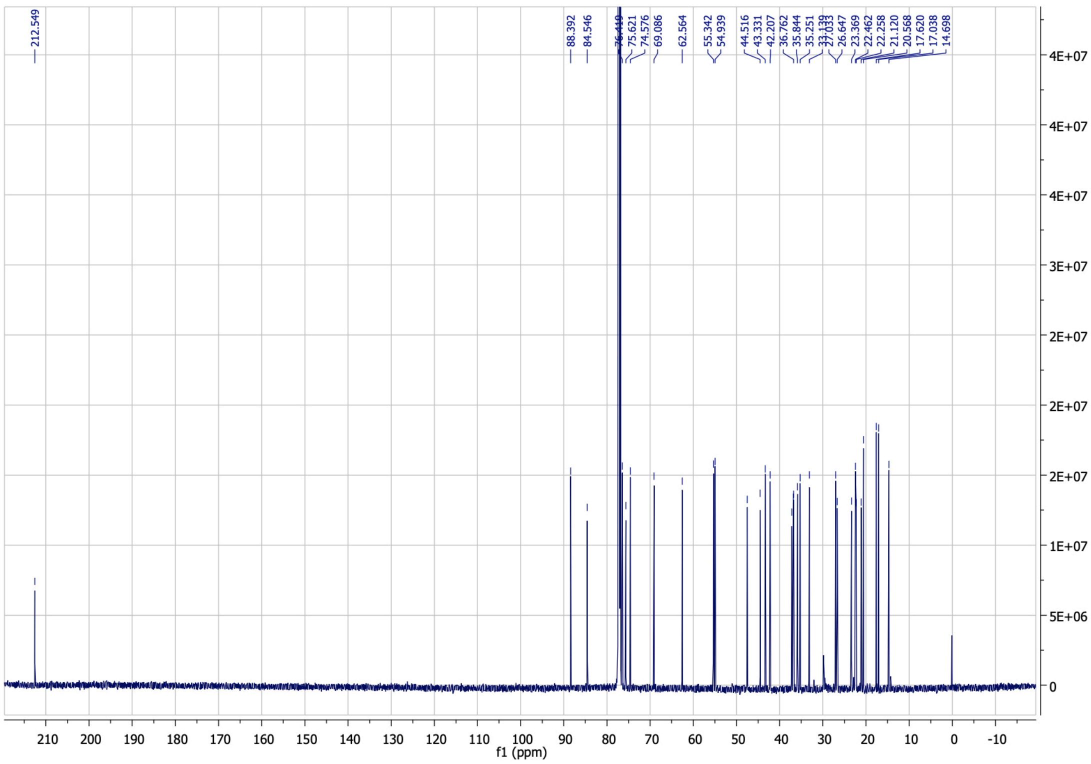

Figure S5. ^{13}Carbon NMR of Neviotin A. ^{13}Carbon NMR spectrum measured in $CDCl_3$.

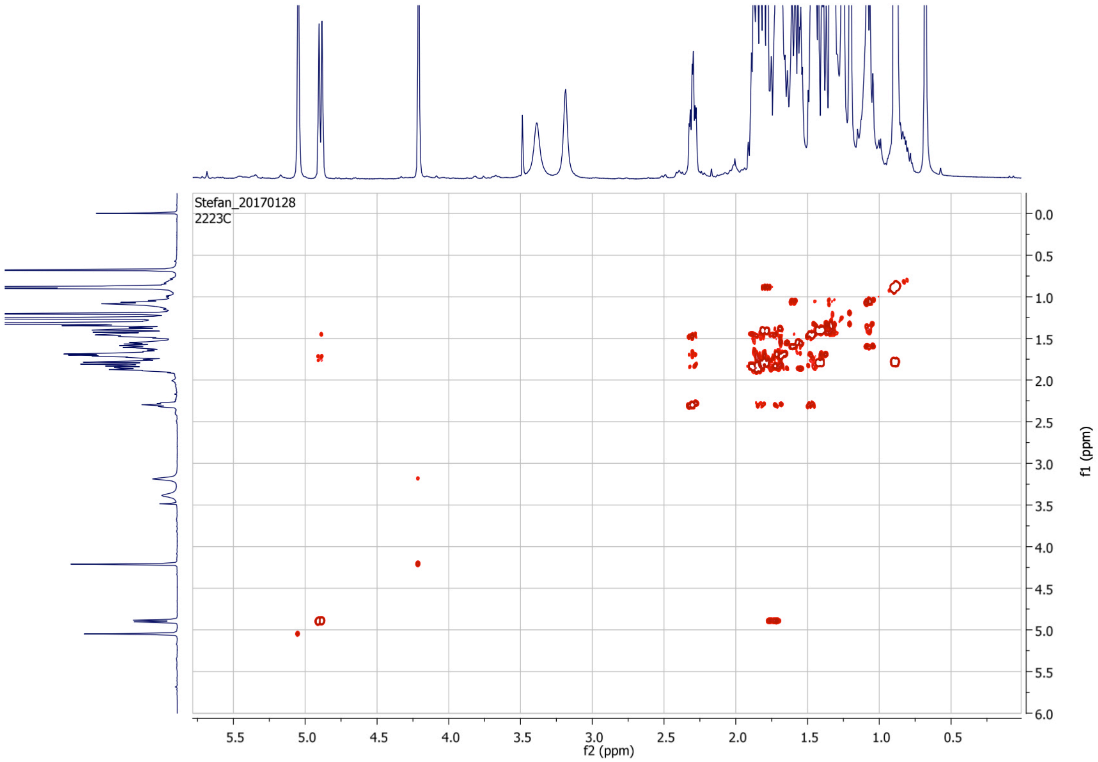

Figure S6. Neviotin A structure analysis by NMR. COSY-spectrum measured in $CDCl_3$.

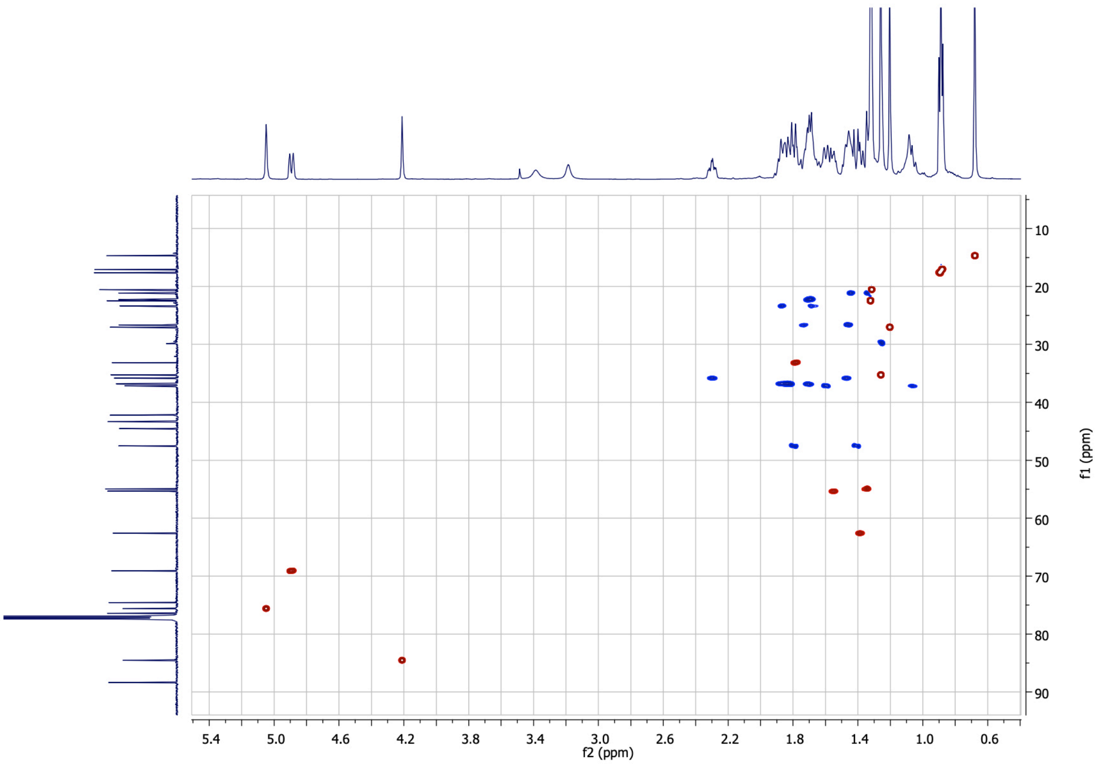

Figure S7. Neviotin A structure analysis by NMR. Heteronuclear Single Quantum Coherence (HSQC)-spectrum measured in $CDCl_3$.

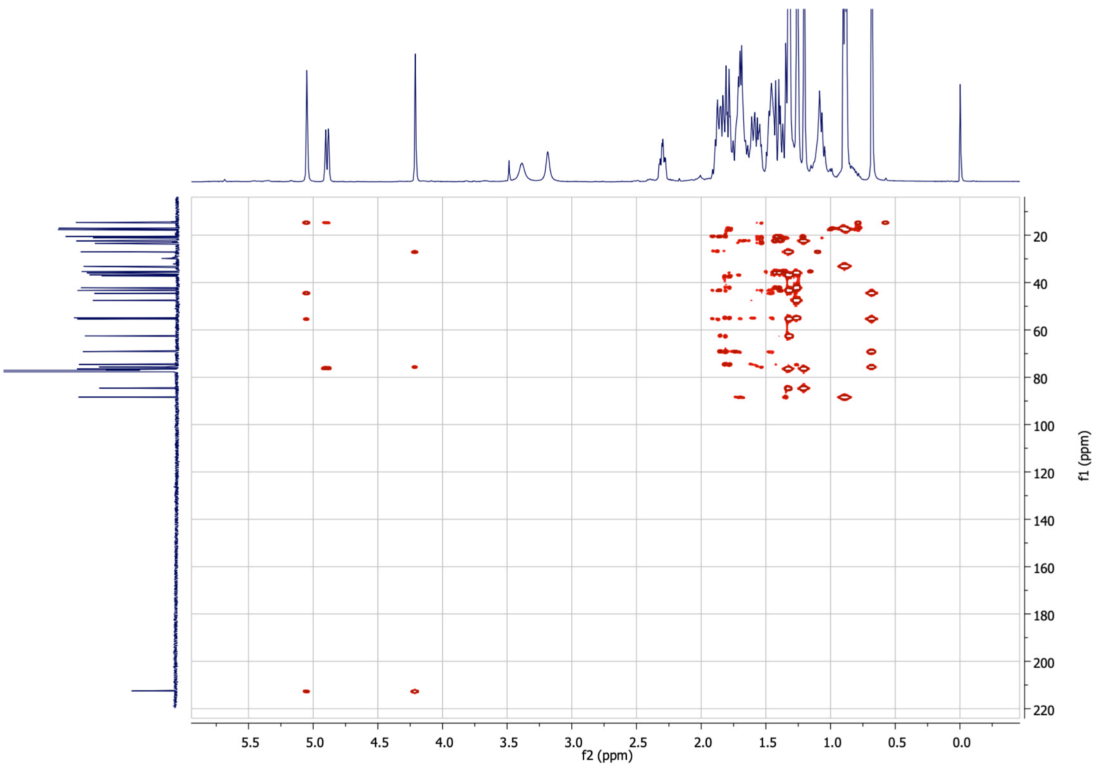

Figure S8. Neviotin A structure analysis by NMR. Heteronuclear Multiple Bond Correlation (HMBC)-spectrum measured in $CDCl_3$.

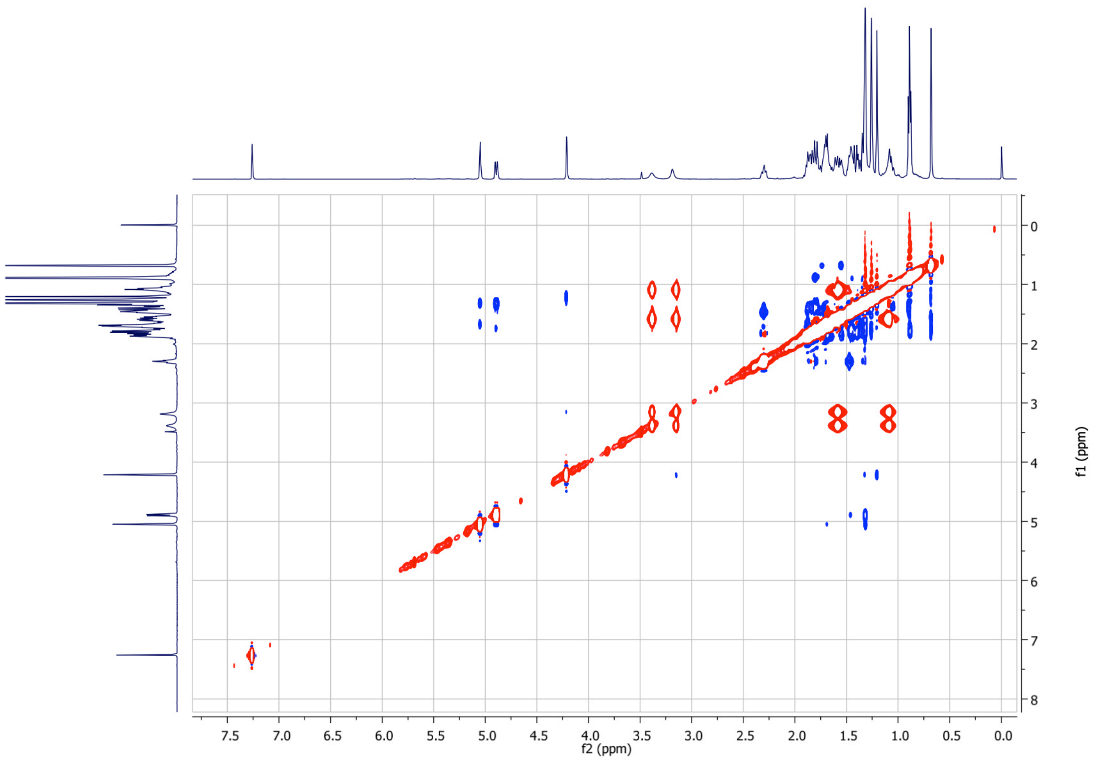

Figure S9. Neviotin A structure analysis by NMR. Nuclear Overhauser Effect Spectroscopy (NOESY)-spectrum in $CDCl_3$.

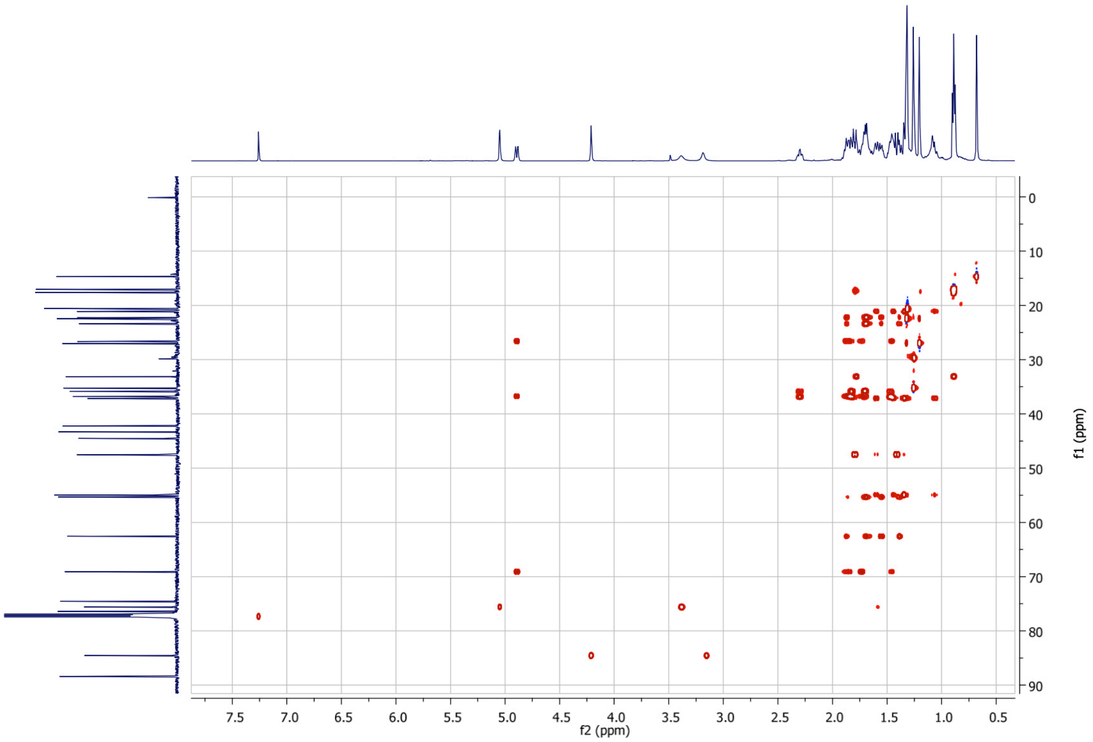

Figure S10. Neviotin A structure analysis by NMR. Heteronuclear Single Quantum Coherence Total Correlation Spectroscopy (HSQC-TOCSY)-spectrum in $CDCl_3$.

Table S1. ^{1}H and ^{13}C NMR data of Neviotin A. NMR data of Neviotin A measured in $CDCl_3$ or DMSO-d_6.

Position	^{1}H NMR (600 MHz, $CDCl_3$)	^{1}H NMR (600 MHz, DMSO-d_6)	^{13}C NMR (151 MHz, $CDCl_3$) δ_C in ppm	^{13}C NMR (151 MHz, DMSO-d_6) δ_C in ppm
2	---	---	76.4	75.6
3	4.21 *s*, 1H	3.94 *d* (4.6), 1H	84.6	83.5
3-O**H**	3.19 *br s*, 1H	6.15 *d* (4.6), 1H	---	---
4	---	---	212.6	213.3
5	5.05 *s*, 1H	4.94 *d* (7.2), 1H	75.6	74.7
5-O**H**	3.39 *br s*, 1H	4.05 *d* (7.2), 1H		
6	---	---	44.5	43.3
7	4.90 *br dd* (12.9; 2.7), 1H	4.85 *dd* (13.0; 3.0), 1H	69.1	68.1
8	Ha 1.73 *m*, Hb 1.46 *m*	Ha 1.58 *m*, Hb 1.27 *m*	26.7	26.4
9	1.84 *m*, 2H	1.73 *m*, 2H	36.8	36.5
10	---	---	43. 3	41.8
11	1.58 *m*, 1H	1.39 *dd* (12.9, 7.2), 1H	55.3	55.1
12	Ha 1.87 *m*, Hb 1.69 *m*	Ha 1.66 *m*, Hb 1.59 *m*	23.4	23.1
13	Ha 1.44 *m*, Hb 1.35 *m*	Ha 1.73 *m*, Hb 1.49 *m*	21.1	21.6
14	1.38 *m*, 1H	1.24 *m*, 1H	62.6	62.4
15	---	---	74.6	72.7
15-O**H**		3.26 *s*, 1H		
16	Ha 1.59 *m*, Hb 1.06 *m*	Ha 1.49 *m*, Hb 0.96 *m*	37.2	36.6
17	1.70 *m*, 2H	1.27 *m*, 2H	22.3	20.8
18	1.34 *m*, 1H	1.29 *m*, 1H	54.9	53.6
19	---	---	88.4	86.2
19-O**H**		3.46 *s*, 1H		
20	Ha 1.84 *m*, Hb 1.70 *m*	1.60 *m*, 2H	36.9	36.1
21	Ha 2.30 *td* (12.1; 4.9), Hb 1.47 *m*	Ha 2.37 *m*, Hb 1.27 *m*	35.8	35.3
22	---	---	42.2	42.6

23	Ha 1.79 *m*,<br>Hb 1.41 *m*	Ha 1.67 *m*,<br>Hb 1.26 *m*	47.5	47.0
24	1.21 *s*, 3H	1.04 *s*, 3H	27.0	26.7
25	1.32 *s*, 3H	1.17 *s*, 3H	22.5	22.4
26	0.68 *s*, 3H	0.56 *s*, 3H	14.7	15.3
27	1.32 *s*, 3H	1.19 *s*, 3H	20.6	20.0
28	1.78 *m*, 1H	1.62 *m*, 1H	33.1	32.9
29	0.90 *d* (7.2),<br>3H	0.80 *d* (6.1),<br>3H	17.6	17.2
30	0.88 *d* (7.2),<br>3H	0.79 *d* (6.1),<br>3H	17.0	17.8
31	1.26 *s*, 3H	1.20 *s*, 3H	35.3	35.3

Table S2. List of upregulated (red text) and downregulated (green text) genes in MCF-7 cells exposed to sample C and Neviotin A analysed by qPCR array experiment.

Sample C

Gene Symbol	Plate Position	Fold Regulation
AKT1	A03	1.22
BAX	A09	1.32
BCL2L1	B01	1.3
BCL2L2	B04	3.16
BIRC5	B10	8.34
CD70	D06	1.78
CRADD	D10	1.47
CYCS	D11	1.22
DIABLO	E02	1.35
IGF1R	E08	1.39
LTBR	E11	1.35
TNFRSF10B	F09	1.39
TNFRSF1B	F12	1.52
TNFRSF21	G01	1.72
ABL1	A01	-1.48
BAG3	A07	-1.42
BCL2A1	A12	-1.54
BID	B06	-1.35
CASP2	C07	-1.23
CASP6	C11	-1.44
FADD	E03	-1.37
FAS	E04	-1.31
NFKB1	F02	-1.21
NOL3	F04	-1.62
TNFRSF1A	F11	-12.38
TP73	G08	-1.27

Neviotin A

Gene Symbol	Plate Position	Fold Regulation
ABL1	A01	4.44
AIFM1	A02	1.66
BAG3	A07	5.82
BAK1	A08	2.97
BCL2A1	A12	5.62
BCL2L10	B02	8.17
BCL2L11	B03	3.34
BID	B06	3.30
BIRC2	B08	1.30
BIRC3	B09	2.21
BIRC5	B10	8.35
BIRC6	B11	1.80
BRAF	C03	1.63
CASP1	C04	11.24
CASP10	C05	3.14
CASP4	C09	1.39
CASP7	C12	4.66
CASP9	D02	4.32
CD27	D03	13.75
CD40LG	D05	6.46
CIDEA	D08	13.56
CYCS	D11	9.01
DFFA	E01	25.30
FAS	E04	8.70
GADD45A	E06	2.18
HRK	E07	2.38
IL10	E09	2.27
LTA	E10	7.90
NOD1	F03	2.07
PYCARD	F05	2.97
RIPK2	F06	1.71
TNFRSF10A	F08	1.59
TNFRSF11B	F10	16.24
TNFSF10	G04	9.20
TNFSF8	G05	3.12
TP53BP2	G07	1.55
TP73	G08	2.57
TRADD	G09	1.68
TRAF3	G11	6.50
AKT1	A03	-7.62
APAF1	A04	-2.08
BAD	A05	-7.25
BAX	A09	-4.62
BCL2	A11	-2.28
BCL2L1	B01	-5.69
BNIP2	B12	-1.33
BNIP3	C01	-1.66
BNIP3L	C02	-1.24
CASP2	C07	-2.79
CD40	D04	-1.41
CD70	D06	-1.35
CIDEB	D09	-2.99
DAPK1	D12	-5.85
DIABLO	E02	-2.55
FADD	E03	-4.05
IGF1R	E08	-5.42
LTBR	E11	-4.66
NFKB1	F02	-2.25
TNF	F07	-2.83
TNFRSF10B	F09	-1.42
TNFRSF1A	F11	-4.53
TNFRSF1B	F12	-2.53
TNFRSF21	G01	-2.39
TNFRSF25	G02	-1.56
TRAF2	G10	-5.02
XIAP	G12	-1.26

Figure S11. Bioinformatic analysis depicting alterations in the miRNAs-gene network map derived from qPCR array data. (A) miRNAs targeting the upregulated (red) and downregulated (green) genes in sample C treated MCF-7 cells. (B) miRNAs targeting the upregulated (red) and downregulated (green) genes in Neviotin A treated MCF-7 cells.

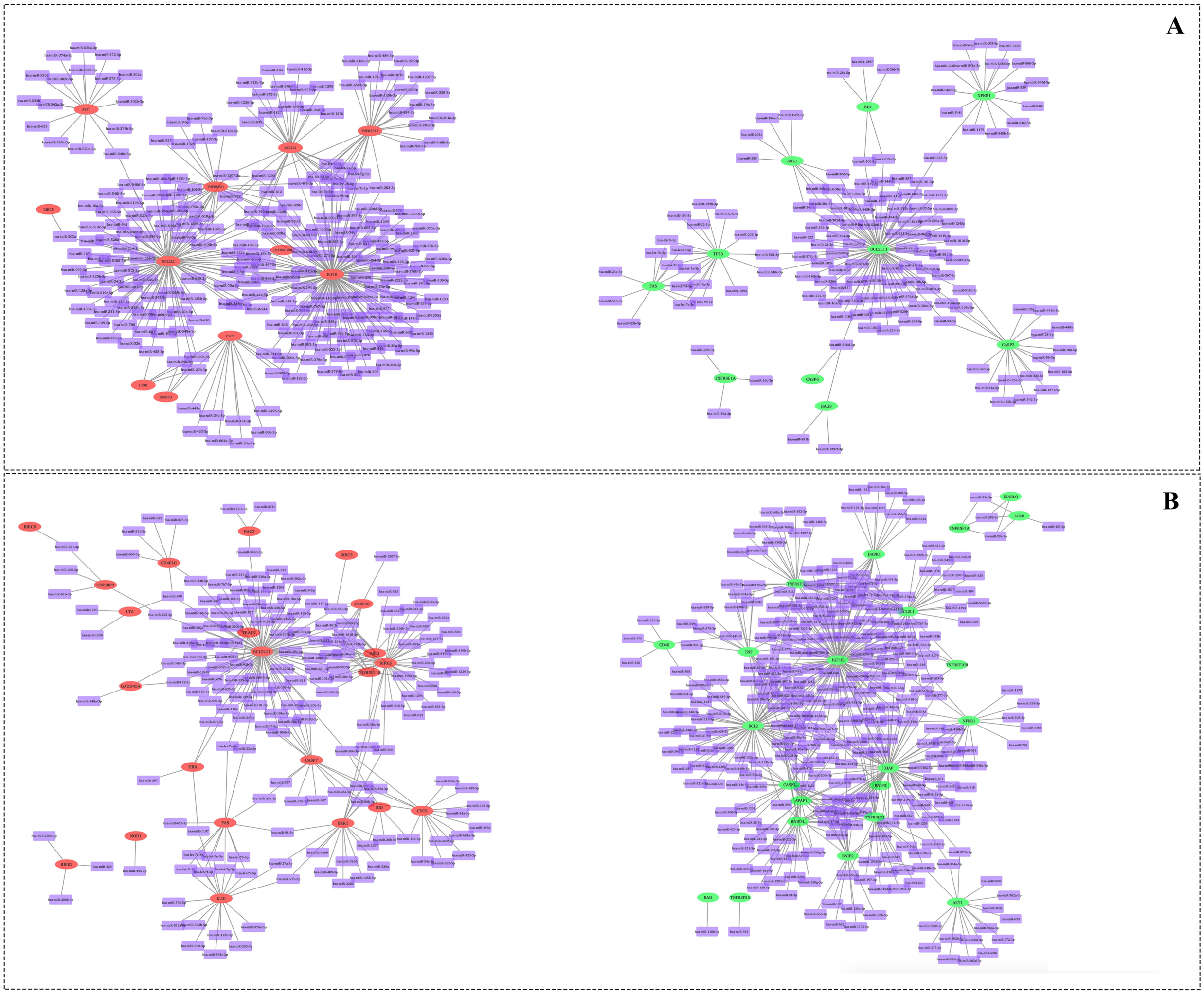

Table S3. Number of miRNAs targeting an individual gene in sample C exposed MCF-7 cells.

Upregulated Genes in Sample C	
Gene names	Number of Associated miRNAs
AKT1	16
BCL2L1	29
BCL2L2	86
BIRC5	1
CYCS	15
DIABLO	3
IGF1R	114
LTBR	4
TNFRSF10B	3
TNFRSF1B	28
TNFRSF21	27

Downregulated Genes in Sample C	
Gene Names	Number of Associated miRNAs
ABL1	9
BAG3	3
BCL2L11	80
BID	5
CASP2	18
CASP6	1
FAS	12
NFKB1	16
TNFRSF1A	3
TP53	17

Table S4. Number of miRNAs targeting an individual gene in neviotin A-exposed MCF-7 cells.

Upregulated Genes in Neviotine A	
Gene Names	Number of Associated miRNAs
ABL1	9
BAG3	3
BAK1	17
BCL2L11	83
BID	5
BIRC3	1
BIRC5	1
BIRC6	38
CASP10	7
CASP7	15
CD40LG	6
CYCS	15
FAS	12
GADD45A	3
HRK	4
IL10	18
LTA	3
NOD1	1
RIPK2	3
TNFRSF11B	5
TP53BP2	4
TRAF3	7

Downregulated Genes in Neviotine A	
Gene Names	Number of Associated miRNAs
AKT1	16
APAF1	20
BAD	1
BCL2	64
BCL2L1	30
BNIP2	20
BNIP3	2
BNIP3L	23
CASP2	21
CD40	5
DAPK1	19
DIABLO	3
IGF1R	114
LTBR	4
NFKB1	17
TNF	17
TNFRSF10B	4
TNFRSF1A	3
TNFRSF1B	28
TNFRSF21	26
TNFRSF25	1
XIAP	71
